# Charge order textures induced by non-linear couplings in a half-doped manganite

**DOI:** 10.1038/s41467-021-24026-7

**Published:** 2021-06-18

**Authors:** Ismail El Baggari, David J. Baek, Michael J. Zachman, Di Lu, Yasuyuki Hikita, Harold Y. Hwang, Elizabeth A. Nowadnick, Lena F. Kourkoutis

**Affiliations:** 1 Department of Physics, Cornell University, Ithaca, NY USA; 2 School of Electrical and Computer Engineering, Cornell University, Ithaca, NY USA; 3 School of Applied and Engineering Physics, Cornell University, Ithaca, NY USA; 4 Department of Physics, Stanford University, Stanford, CA USA; 5 Stanford Institute for Materials and Energy Sciences, SLAC National Accelerator Laboratory, Menlo Park, CA USA; 6 Department of Applied Physics, Stanford University, Stanford, CA USA; 7 Department of Materials Science and Engineering, University of California Merced, Merced, CA USA; 8 Kavli Institute at Cornell for Nanoscale Science, Cornell University, Ithaca, NY USA; 9 Present Address: Intel Corp., Hillsboro, OR USA; 10 Present Address: Center for Nanophase Materials Sciences, Oak Ridge National Laboratory, Oak Ridge, TN USA; 11 Present Address: Materials Science and Engineering, Northwestern University, Evanston, IL USA

**Keywords:** Electronic properties and materials, Quantum fluids and solids

## Abstract

The self-organization of strongly interacting electrons into superlattice structures underlies the properties of many quantum materials. How these electrons arrange within the superlattice dictates what symmetries are broken and what ground states are stabilized. Here we show that cryogenic scanning transmission electron microscopy (cryo-STEM) enables direct mapping of local symmetries and order at the intra-unit-cell level in the model charge-ordered system Nd_1/2_Sr_1/2_MnO_3_. In addition to imaging the prototypical site-centered charge order, we discover the nanoscale coexistence of an exotic intermediate state which mixes site and bond order and breaks inversion symmetry. We further show that nonlinear coupling of distinct lattice modes controls the selection between competing ground states. The results demonstrate the importance of lattice coupling for understanding and manipulating the character of electronic self-organization and that cryo-STEM can reveal local order in strongly correlated systems at the atomic scale.

## Abstract (web summary)

In this paper, the authors demonstrate that cryogenic scanning transmission electron microscopy allows for the direct mapping of the local arrangements and symmetries of electronic order, providing a useful method for studying strongly correlated systems. They show this using the example of Nd1/2Sr1/2MnO3, a model charge ordered material.

## Introduction

**Fig. 1:**
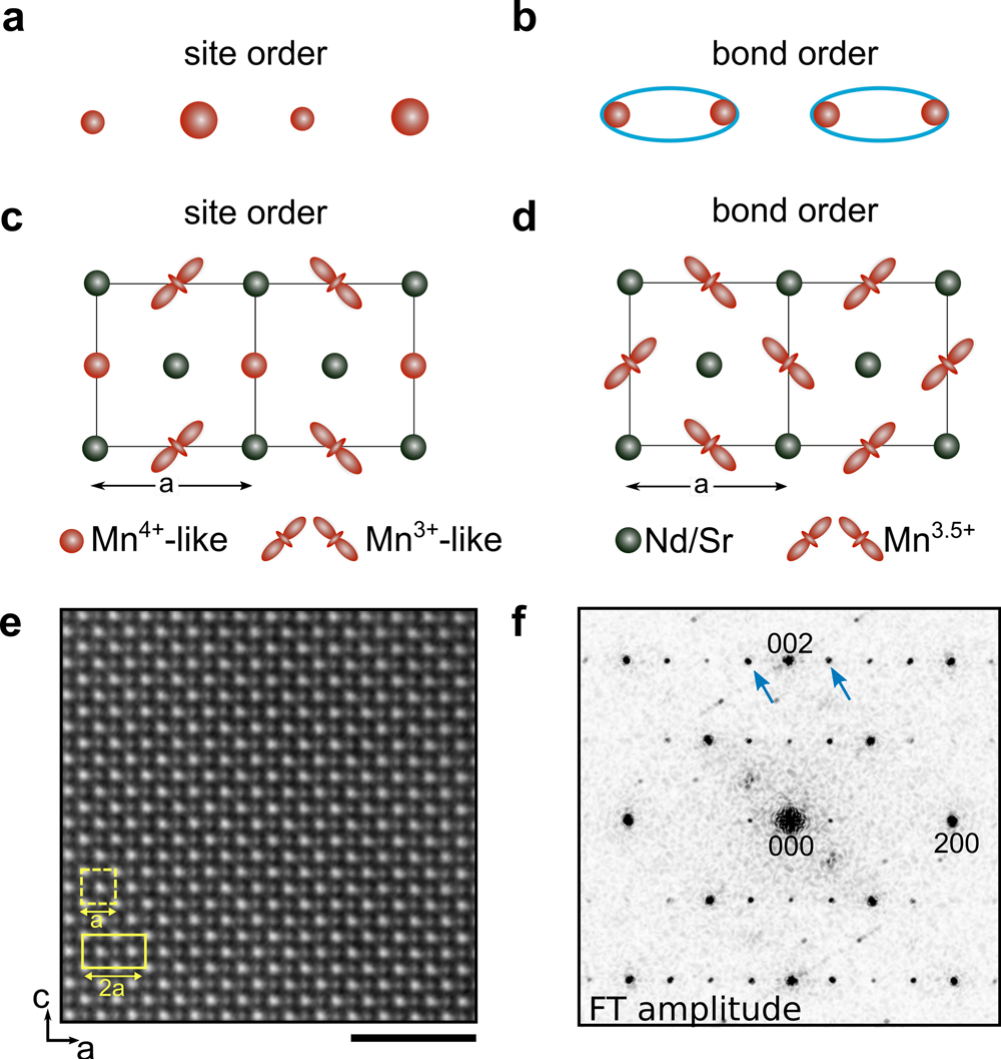
Charge-order supercell. **a** Charge-order modulation centered on sites (red circles). The size of the circle indicates the electronic density on the site. **b** Charge-order modulation centered on the bonds. Ellipses represent stronger, more electron-rich bonds. The sites remain equivalent in density. **c** Prototypical site-centered charge and orbital order model in the half-doped manganites, including orbital order. **d** Alternative bond-centered model in which all Mn sites have the same charge configuration (Mn^3.5+^). In this case, the supercell is generated by the orbital order. **e** High-angle annular dark-field (HAADF) scanning transmission electron microscopy (STEM) projection image at 93 K, where we expect the system to be in the charge-ordered phase. Bright atomic columns represent Nd/Sr sites and dark atomic columns represent Mn sites. The scale bar corresponds to 2 nm. **f** Fourier transform amplitude of the STEM image. At 93 K, superlattice peaks (arrows) with **Q**^*CO*^ = (1/2, 0, 0) are evident, indicating the formation of a twofold superlattice in real space (box in **e**).

Strong interactions between electrons and the atomic lattice often lead to their self-organization into ordered spatial patterns^1–4^. One well-known example is charge ordering, the spatial modulation of the electronic charge density which forms superlattices and governs the properties of many exotic materials, from oxides to transition-metal chalcogenides to charge-transfer salts^5–7^. In general, charge ordering is studied at scales larger than the superlattice (inter-unit-cell structure), with a focus on the average periodicity of the charge modulation or the degree of long-range order^8,9^. On the other hand, the microscopic arrangement at sub-unit-cell length scales (intra-unit-cell structure), such as whether the electrons reside on the atomic sites or bonds (Fig. 1a, b), is significantly more challenging to measure but dictates the symmetry of the system in addition to the mechanism underlying electronic order^10–12^.

Perovskite 3*d* transition-metal oxides are a class of materials in which charge order plays an especially important role, influencing antiferromagnetic order, high-temperature superconductivity, and colossal magnetoresistance^5,13,14^. The strong hybridization between the transition metal (site) and oxygen (bond) in these systems contributes to their rich, intricate electronic structure landscape^15^. In cuprates, for instance, recent bulk measurements suggest that the charge-ordered phase may contain a site-centered modulation in addition to the putative bond-centered *d*-wave modulation^10,16^. A similarly complex and fundamental debate concerns the half-doped manganites, an ideal playground for exploring coupled charge and orbital orders and phase competition^17^. The widely accepted model, proposed over 60 years ago, invokes a site-centered modulation in which electrons localize on manganese sites and form a zig-zag orbital order pattern that doubles the unit cell (Fig. 1c)^18–20^. An alternative bond-centered state has also been proposed^21^; in this case, a charge modulation does not occur so the superlattice is generated from orbital ordering (Fig. 1d). Subsequent experimental studies, however, have not conclusively confirmed this scenario^20,22,23^. The theory further predicts an even more exotic intermediate state which mixes both site and bond characters, leading to the breaking of inversion symmetry through the formation of uncompensated electric dipoles^24,25^. Determining if such a state exists or whether charge order corresponds to a pure site- or bond-centered state remains a fundamental challenge, with broad and urgent implications for other classes of materials.

Manganite compounds exhibit large interactions between the lattice and the electronic degrees of freedom, and so most experimental proposals for site- or bond-centered charge-order models have relied on obtaining the average lattice distortions and crystal symmetry^19,21^. Here, we employ atomic-resolution cryogenic scanning transmission electron microscopy (cryo-STEM) to probe charge ordering by directly mapping picoscale lattice displacements in real space. In previous work, this method revealed the inter-unit-cell variations of incommensurate charge order in an overdoped manganite but did not address the microscopic structure within the superlattice^26^. Here, we extend this approach to directly reveal coexisting intra-unit-cell arrangements of charge order in a half-doped manganite and the role of additional lattice modes in mediating such arrangements. Importantly, our visualizations establish the existence of the intermediate phase, a ground state that had remained undetected in part due to the challenge of disentangling nanoscale domains of pure bond/site order from genuine mixed states in measurements with low spatial resolution. Going further, cryo-STEM imaging and theoretical calculations reveal coupled secondary order parameters that govern the nature of the charge-order phase. These atomic-scale visualizations not only advance our understanding of charge ordering but also reveal a tuning knob for manipulating electronic self-organization via lattice coupling.

## Results

The material we focus on is a Nd_1/2_Sr_1/2_MnO_3_ thin film which was grown using pulsed laser deposition on a (110)-oriented SrTiO_3_ substrate. The particular substrate orientation, which imparts in-plane anisotropy on the film, reproduces the electronic and structural transitions found in the bulk counterpart including the charge-order transition^27–29^. The temperature dependence of the magnetization in this film matches that of previously reported epitaxial films. Figure 1e shows a projection HAADF-STEM image of the crystal along the [010] orientation (in the *Pnma* space group setting) below the charge-order transition temperature (T_*c*_ ~ 150 K). The atomically resolved Nd/Sr columns appear brighter than the Mn columns because the contrast in HAADF scales strongly with the atomic number. Near the crystalline peaks in the Fourier transform amplitude (Fig. 1f), superlattice peaks (blue arrows) appear at low temperatures, indicating the formation of a modulated structure. These are located at **Q**^*CO*^ = (1/2, 0, 0) as expected for charge ordering at half doping. The modulation is unidirectional, however, some regions of the sample exhibit bi-directional modulations. These might arise from the coexistence of small charge-order domains (either in-plane or out-of-plane) within a single crystal twin or from the presence of crystalline twins that establish the direction of the charge-order wavevector. By mapping both the charge order and the crystalline order parameters, we find that the orthogonal charge-order domains are coupled to crystalline twins in the sample (Supplementary Fig. 3).

The exact intra-unit-cell arrangement within charge-order superlattices is key to understanding their microscopic origins and interactions with other electronic phases. In the majority of theoretical treatments of charge-ordered phases, the ground state is discussed in terms of pure electronic degrees of freedom. Site-centered charge order in half-doped manganites, for instance, is described as the alternation of Mn^4+^ and Mn^3+^ species^18,19^; however, the degree of charge disproportionation is much smaller and is better described by Mn valences of 3.5 + *δ* (Mn^4+^-like) and 3.5 − *δ* (Mn^3+^-like) with *δ* ≪ 0.5^20,30^. Measurements of the charge modulation in manganites, whether using resonant X-ray scattering or electron energy loss spectroscopy, have been complicated by the small amplitude of the valence modulation and by the presence of strong lattice distortions^20,30–32^. In fact, the crystal structure in the charge-ordered state undergoes a variety of complex atomic displacements, such as Jahn–Teller and breathing distortions, which alter the bonding network and hence the electronic configuration. Therefore, emergent charge and orbital textures are closely linked to the pattern and symmetry of said displacements^33,34^. For site-centered order, bulk X-ray or neutron structural data suggest that the crystal adopts *P*2_1_/*m* space group symmetry with bond distortions consistent with charge localization on the Mn sites^19,20,22^. The experimental report for the bond-centered model, on the other hand, found a different space group symmetry (*Pnm*2_1_) and a distinct displacement pattern associated with it^21^. The ability to probe intra-unit-cell lattice distortions can therefore determine or even reveal novel ground states.

**Fig. 2:**
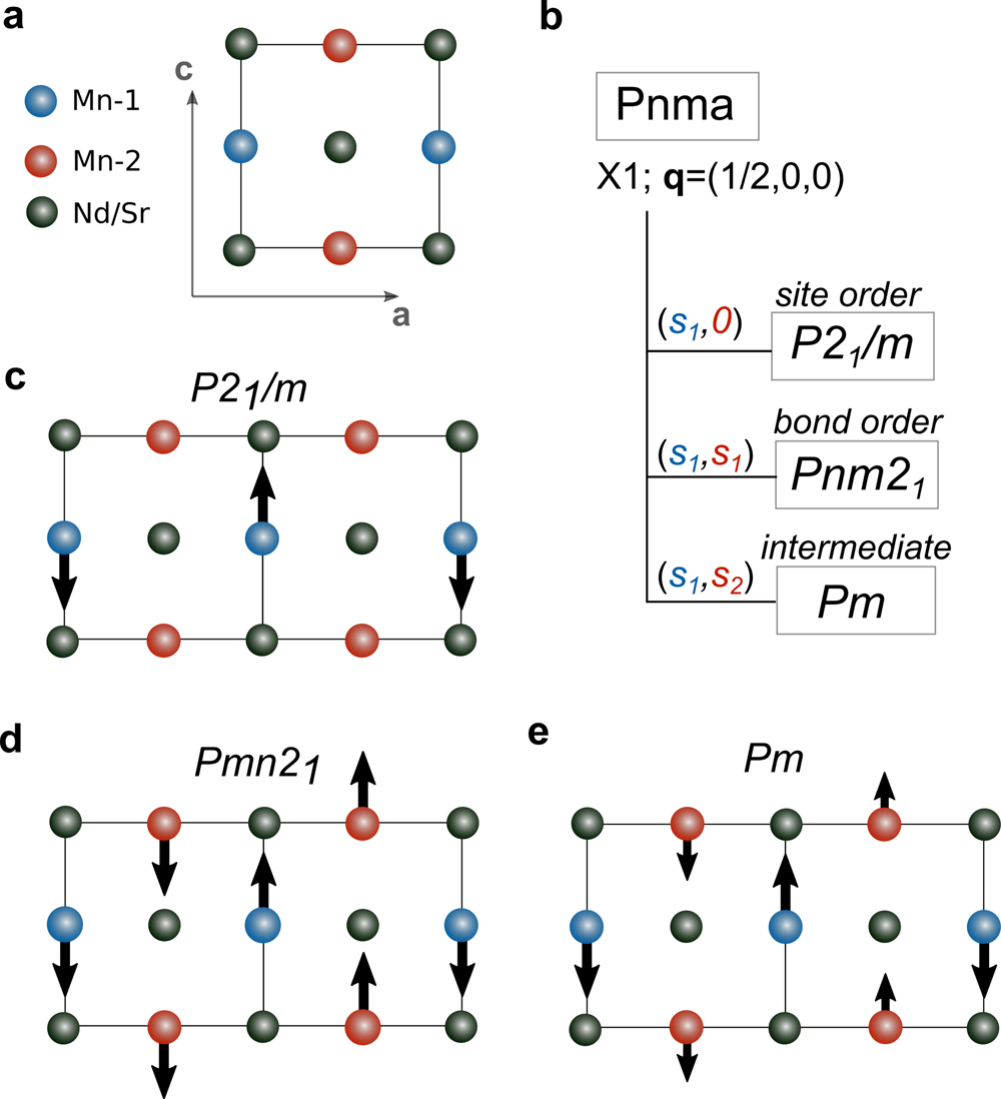
The symmetry of distorted structures. **a** High-symmetry unit cell with two Mn sublattices. **b** Group-subgroup relations for displacements with the wavevector **q** = (1/2, 0, 0) and irreducible representation X1. The X1 order parameter is two-dimensional with each dimension corresponding to a Mn sublattice. **c**–**e** Displacement patterns for the site-centered (*s*_1_,*s*_2_ = 0), bond-centered (*s*_1_,*s*_2_ = *s*_1_), and intermediate (*s*_1_,*s*_2_ ≠ *s*_1_) phase, respectively.

To firmly connect patterns of atomic displacements to the reported crystal symmetries and hence to the models of electronic order, we first explore the possible distorted structures that emerge from the high-symmetry phase (space group *Pnma*) using group theory. Any distorted structure must double the unit cell, therefore we require atomic displacements whose wavevector is **q** = (1/2, 0, 0). The relevant distortion consistent with this requirement is the X1 displacement mode, or irreducible representation (irrep), which affects two inequivalent Mn sublattices in the high-symmetry unit cell (see Supplementary Information section 4). In other words, the X1 irrep is two-dimensional with the first dimension corresponding to the first Mn sublattice (Mn-1 in blue) and the second dimension to the second Mn sublattice (Mn-2 in red), as shown in Fig. 2a. If X1 displacements, which consist of a complex set of atomic distortions including the transverse Mn displacements shown in Fig. 2, affect only the first sublattice, the resulting crystal structure has *P*2_1_/*m* symmetry, which is consistent with site-centered order (Fig. 2b, c). If they occur in both sublattices and with equal magnitude (Fig. 2b, d), the resulting crystal structure has *Pnm*2_1_ symmetry, which matches that of bond-centered order. The third structure with *Pm* symmetry can be obtained by having displacements on both sublattices but with different magnitudes (Fig. 2b, e). Such an intermediate state combines aspects of both site and bond order, however, experimental refinements of atomic positions in this symmetry have not been performed so far. The group theory analysis thus shows that the pattern of Mn displacements uniquely defines the character of the charge-ordered phase, without resorting to measurements of the electronic charge.

**Fig. 3:**
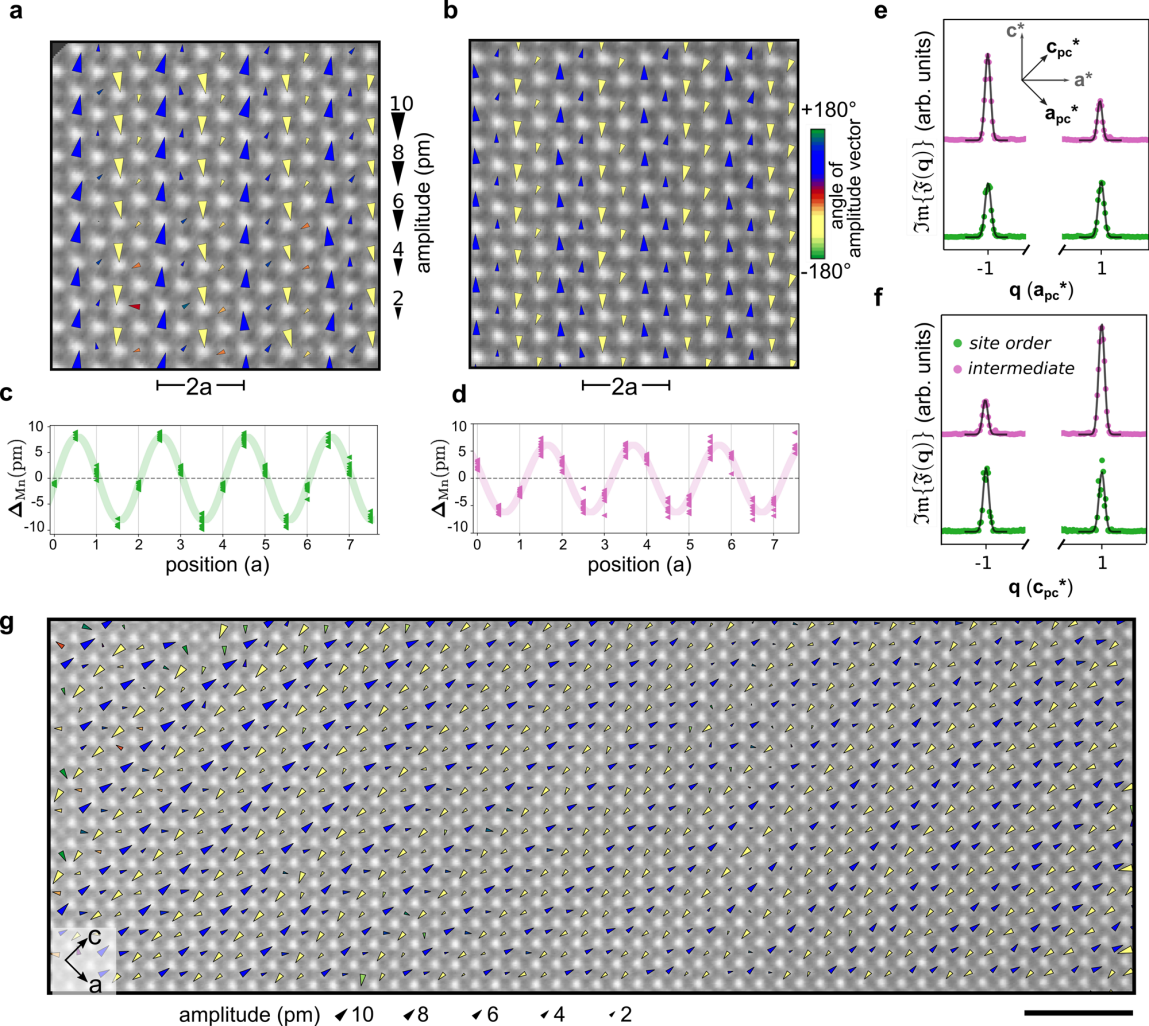
Nanoscale coexistence of site order and noncentrosymmetric intermediate order. **a** Map of periodic lattice displacements of manganese atomic columns (**Δ**_Mn_). The area of the arrows scales with the amplitude of the distortion and the color represents the angle relative to the wavevector direction. In this case, the colors (blue/yellow) indicate a transverse polarization of the displacements (+90°/−90°). The displacement pattern within the sub-region is consistent with site-centered order; one Mn sublattice shows large displacement amplitude while the other shows much smaller displacements. **b** Periodic lattice displacement map in another sub-region shows displacements on both sublattices. Further, the sublattice displacement amplitudes are not equal which indicates intermediate order. **c** Mn displacements (dots) and sinusoidal envelope function (line) in the site-centered phase. **d** Mn displacements (dots) and sinusoidal envelope function (line) in the intermediate phase. **e**, **f** Line cuts across the imaginary part of local Fourier transforms, ${\mathfrak{Im}}\{{\mathfrak{F}}({\bf{q}})\}$, along the pseudocubic directions ${{\bf{a}}}_{{\bf{pc}}}^{* }$ and ${{\bf{c}}}_{{\bf{pc}}}^{* }$. The site-centered phase (green) maintains ±**q** symmetry whereas the intermediate phase (magenta) shows clear differences between the ±**q** peaks, an indication that the latter phase breaks inversion symmetry. **g**, Large field-of-view map of periodic Mn displacements shows a transition from site-centered order (left side) to intermediate order (right side). The scale bar corresponds to 2 nm.

We now pinpoint the underlying ordering model in Nd_1/2_Sr_1/2_MnO_3_ by mapping the picoscale lattice degrees of freedom using HAADF-STEM at low temperature. A reference image lacking the periodic modulation is generated by removing the contribution of the **Q**^*CO*^ superlattice peaks in the Fourier transform^26,32^. The displacements are then extracted by mapping the atomic positions in the original image and the reference image. Figure 3a shows a STEM image overlaid with arrows indicating Mn column displacements. The area of the triangle denotes the magnitude of displacement and the color represents the angle of the displacement relative to the wavevector. The dominant displacements have transverse polarization and generate a twofold superlattice.

The displacement pattern shown in Fig. 3a matches site-centered order. The first Mn sublattice has a large displacement amplitude (8.2(9) pm) while the second has a comparatively negligible amplitude (1.3(6) pm), similar to the group theory prediction of site-centered order. The manganese displacements as a function of position, **r**, are sinusoidal (Fig. 3c) and may be described by ${{\mathbf{\Delta }}}_{\text{Mn}}({\bf{r}})={\bf{A}}\sin ({{\bf{Q}}}^{CO}\;.\;{\bf{r}}+\phi )$ where **Δ**_Mn_(**r**), **A**, **Q**^*CO*^, and *ϕ* are the displacement, amplitude vector, wavevector, and the phase, respectively. The phase determines the centering of the modulation relative to the Mn sites, with site- and bond-centered order corresponding to *ϕ* = *nπ* (*n* is an integer) and *ϕ* = *nπ*/2 (*n* is an odd integer), respectively. A deviation of the phase from these two limits corresponds to a state intermediate between site and bond order. Fitting the displacements in the region shown in Fig. 3a further supports that the modulation is predominantly site-centered with *ϕ* = 0.04(1) *π*. From this measurement, we thus confirm the presence of the prototypical site-centered state.

Remarkably, we discover that within the same sample another region has a distinct displacement pattern: prominent Mn displacements occur in both Mn sublattices (Fig. 3b). Importantly, the displacements have different amplitudes on each sublattice, unlike the predicted pattern for purely bond-centered order. By comparing to the group theory analysis of displacement patterns, the observed structure is consistent with a state which is intermediate between pure site and bond order (*Pm* structure). Figure 3d shows the aggregated Mn displacements and their sinusoidal envelope. The intermediate structure is readily apparent since the maximum of the sinusoidal function is not centered on Mn sites nor exactly at the middle. The phase is *ϕ* = 0.35(1) *π* and the mean displacement amplitudes on the first and second sublattices are 2.9(9) pm and 5.8(7) pm, respectively. Given that STEM is a projection imaging technique, we ruled out the possibility that the intermediate state merely reflects a projection of stacked site-centered states along the beam direction. This is achieved by analyzing both the contrast variations in the image and the patterns of displacements (see Supplementary Information section 3 for more details). Cryo-STEM mapping of lattice displacements, therefore, provides direct evidence for intermediate charge order in manganites, an observation with implications for other oxides in which the exact character of charge order remains unresolved.

An intriguing consequence of the overlap of the site- and bond-centered order is that additional crystal symmetries may be broken^24^. In oxides or charge-transfer salts, intermediate order is predicted to break inversion symmetry due to the formation of uncompensated dipoles, motivating proposals for unconventional ferroelectricity emerging from electronic order^24,25,35–38^. To determine whether the intermediate phase is indeed noncentrosymmetric, we compute local complex-valued Fourier transforms of the site-centered and intermediate regions. The imaginary part, ${\mathfrak{Im}}\{{\mathfrak{F}}({\bf{q}})\}$, is sensitive to the odd component of the atomic-resolution image and hence to the breaking of inversion symmetry^39^. For a noncentrosymmetric structure, we, therefore, expect the crystalline Bragg peaks at ±**q** positions to be inequivalent. Figure 3e, f shows integrated line cuts through ${\mathfrak{Im}}\{{\mathfrak{F}}({\bf{q}})\}$ along the pseudocubic ${{\bf{a}}}_{pc}^{* }$ and ${{\bf{c}}}_{pc}^{* }$ directions, respectively. In the site-centered phase (green), the imaginary component of the Bragg peaks has the same amplitude at ±**q** coordinates for both pseudocubic directions, which indicates that inversion symmetry is maintained. In contrast, there is a significant difference in the intermediate case (magenta), confirming that inversion symmetry is broken in that region. The group theory analysis also tells a consistent story with the intermediate phase having a noncentrosymmetric (*Pm*) structure. While intermediate charge order can in principle be detected through the switching of bulk polarization, the finite conductivity and nanoscale spatial inhomogeneity of manganites have precluded such an approach^25^. Atomic-scale cryo-STEM imaging, on the other hand, confirms the existence of intermediate charge ordering and the resultant breaking of inversion symmetry.

Another key insight from these local visualizations is that ground states with distinct symmetries may coexist within the same system. Both Fig. 3a, b are taken from the same field-of-view image (Fig. 3g), in which the site-centered phase (left side) transforms into the intermediate phase (right side) over a few unit cells. Within this field of view, strain variations are likely too small to govern the nature of the charge-order ground state (Supplementary Fig. 4a–c). Instead, a possible origin for the coexistence is the presence of quenched disorder which is known to lead to nanoscale phase coexistence in correlated oxides. In the case of the Nd_1/2_Sr_1/2_MnO_3_ sample studied here, we detect local but the short-range ordering of the A-site cations (Nd/Sr), which manifests in the Fourier transform as broad half-order peaks along the pseudocubic direction ${{\bf{a}}}_{{\bf{pc}}}^{* }$. The local amplitude of the cation ordering (Supplementary Fig. 4d) confirms its short-range character, but, more importantly, shows that the quenched disorder occurs in both the site-centered and intermediate phase. We surmise that the coexistence emerges from deformations of the phase of the charge-order state, to accommodate both the pinning to the disorder and the energy cost of deviating from a constant phase. Such scenario is even more likely if the energies of the two charge-order states are comparable, highlighting the long-standing difficulty in determining the true ground state away from the disorder-free limit.

**Fig. 4:**
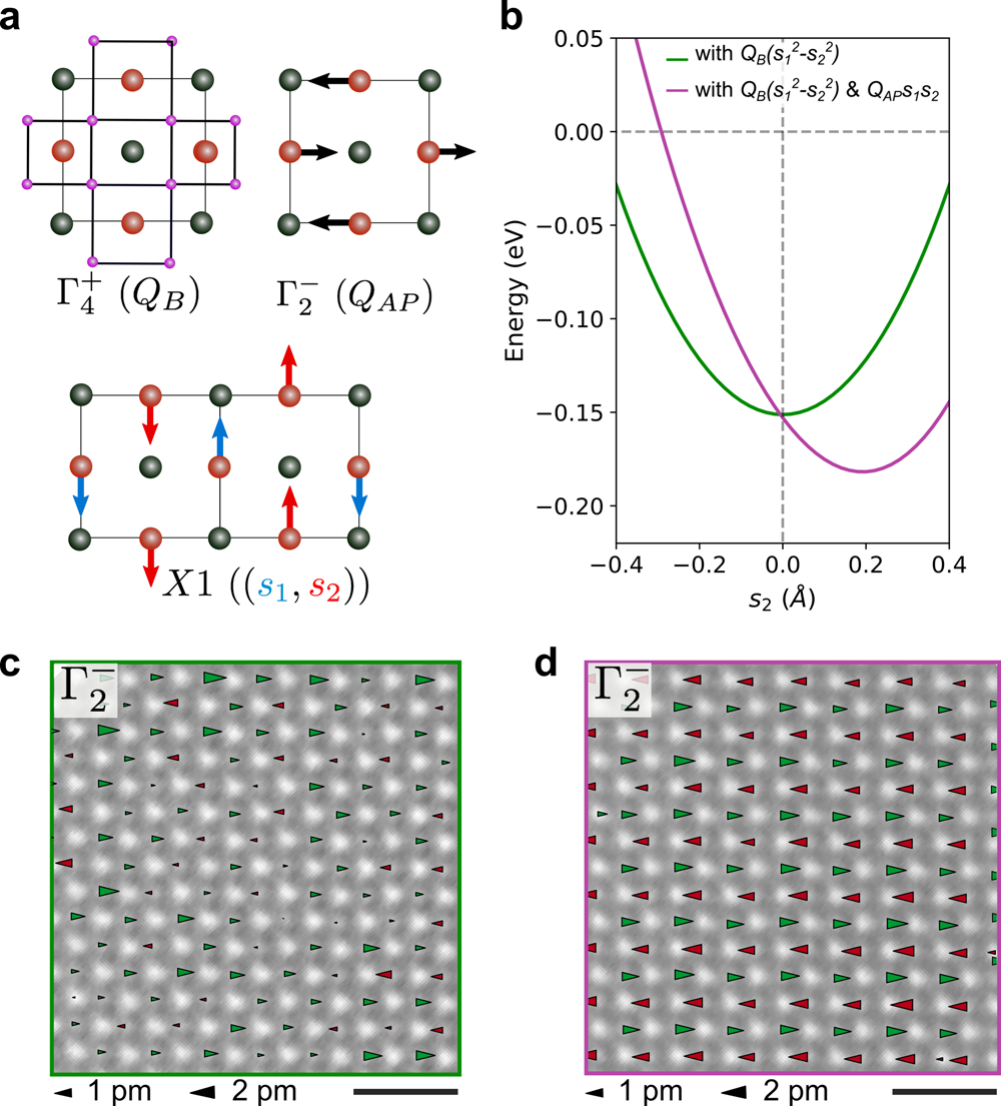
Nonlinear lattice coupling determines the charge-order ground state. **a** Displacement patterns for the ${{{\Gamma }}}_{4}^{+}$ mode (oxygen breathing distortion), the ${{{\Gamma }}}_{2}^{-}$ mode (Mn antipolar displacement), and the X1 mode. The purple dots represent the oxygen atoms. **b** One-dimensional plot of the full Landau free energy (Eq. (3) in Supplementary Information section 5) as a function of the amplitude of *s*_2_. The linecut is taken along the (0, *s*_2_) axis and through the *s*_1_ value that minimizes the energy. When the first coupling term is included and the second term is suppressed (green) the minimum energy is located at (*s*_1_ ≠ 0, *s*_2_ = 0) indicating that site-centered order is favorable. If both coupling terms are present (magenta) both *s*_1_ and *s*_2_ are finite but not equal indicating that intermediate order is favorable. The values for the coefficients in the Landau free energy and the *Q*_*B*_/*Q*_*AP*_ amplitudes are listed in the Supplementary Information section 5. The amplitude of *s*_2_ (in Å) is with respect to a 40-atom DFT supercell. **c**, **d** Atomic-scale maps of the ${{{\Gamma }}}_{2}^{-}$ Mn displacements in the site-centered and intermediate region, respectively. The largest arrows correspond to ~2 pm. The displacements are disordered in the former and coherent in the latter, in agreement with the Landau theory predicting that the ${{{\Gamma }}}_{2}^{-}$ mode favors X1 displacements on both sublattices. Both scale bars correspond to 1 nm.

Informed by the atomic-scale evidence for site and intermediate orders, we next examine the possible origin of these two states. While we have focused on the X1 displacements so far, the low-temperature phase may also contain additional structural responses that are allowed by symmetry (Fig. 4a). In particular, a ${{{\Gamma }}}_{4}^{+}$ mode, which appears as an oxygen breathing distortion, and a ${{{\Gamma }}}_{2}^{-}$ mode, which appears as antipolar displacements of Mn atoms, are such symmetry-allowed responses (see Supplementary Information section 4). To understand their role, we expand the Landau free energy about the high-symmetry phase up to fourth order in powers of the ${{{\Gamma }}}_{4}^{+}$, ${{{\Gamma }}}_{2}^{-}$, and X1 displacement mode amplitudes (see Eq. (3) in the Supplementary Information section 5 for the expansion). Significantly, this expansion contains two relevant third-order coupling terms:

1 $${F}_{3}={g}_{bs}{Q}_{B}({s}_{1}^{2}-{s}_{2}^{2})+{g}_{as}{Q}_{AP}{s}_{1}{s}_{2}$$

where *Q*_*B*_, *Q*_*AP*_, and (*s*_1_, *s*_2_) are the amplitudes of the ${{{\Gamma }}}_{4}^{+}$, ${{{\Gamma }}}_{2}^{-}$, and X1 distortions, respectively. From density functional theory (DFT) calculations (see Supplementary Information section 5), we find that the *g*_*bs*_ and *g*_*as*_ coefficients are negative, which indicates that the coupling terms lower the energy. We, therefore, propose that the third-order nonlinear couplings between the Γ and X1 modes provide a mechanism for favoring one ground state over another.

We now consider the implications of the two coupling terms in Eq. (1). The first term stabilizes the breathing distortion and lowers the energy the most in the site-centered phase where the X1 displacements are (*s*_1_ ≠ 0, *s*_2_ = 0). The second term lowers the energy the most in the presence of the antipolar distortions and the bond-centered phase which has X1 displacements (*s*_1_, *s*_2_ = *s*_1_). In both of these cases, where only one of the coupling terms is nonzero, we obtain a pure solution (site or bond). If both coupling terms are nonzero, the lowest energy is achieved with the X1 amplitude equal to (*s*_1_, *s*_2_ ≠ *s*_1_), which corresponds to intermediate order. To illustrate, we show one-dimensional line cuts across the minimum of the Landau energy in Fig. 4b, having set the coefficients and amplitudes to the DFT-calculated values (see Supplementary Information section 5 and Supplementary Tables II and III). If we keep the first coupling term and suppress the second, the free energy is minimized by having (*s*_1_ ≠ 0, *s*_2_ = 0) in the X1 amplitude, indicating that site-centered order is favorable. When both coupling terms are present, the minimum of the free energy shifts away from (*s*_1_, 0) to (*s*_1_, *s*_2_ ≠ 0), thus stabilizing the intermediate phase through a nonlinear mechanism. A more complete picture of the energy dependence on the various coupling terms can be found in 2D plots of the energy surfaces (Supplementary Fig. 11). Based on this theory, a key prediction is that the antipolar ${{{\Gamma }}}_{2}^{-}$ displacements are absent (present) in the experimentally observed site-centered (intermediate) phase.

To test this prediction, we visualize the spatial interplay between the antipolar Mn displacements and the character of the charge-ordered ground state. Figure 4c, d show maps of the antipolar ${{{\Gamma }}}_{2}^{-}$ Mn displacements in the regions containing site and intermediate order, respectively. In the region with site-centered order, the ${{{\Gamma }}}_{2}^{-}$ mode is disordered, lacking any clear pattern of antipolar distortions. In the region with intermediate order, however, this mode is coherent and relatively strong, in agreement with the Landau theory prediction.

## Discussion

Untangling the roles of electronic and structural mechanisms of charge ordering continues to be a major challenge. While initial predictions proposed purely electronic mechanisms behind the formation of the various charge-ordered states in manganites^24,40^, our observations show that lattice degrees of freedom play a more complex role than appreciated. For instance, textbook charge disproportionation on Mn sites is equivalent to the ${{{\Gamma }}}_{4}^{+}$ breathing distortion from both chemical and symmetry perspectives. The calculations herein, however, show that this charge disproportionation mode raises the energy of the system on its own and that it must couple to additional modes with large structural responses in order to materialize. Similarly, the emergence of the exotic intermediate phase relies on the softening of the ${{{\Gamma }}}_{2}^{-}$ displacements, further underscoring a critical role of the lattice.

These unusual lattice couplings not only challenge our microscopic understanding of the origin of charge ordering but also provide a mechanism for manipulating the character of electronic order and its associated electronic properties. In the case of the intermediate phase, an exciting theoretical prospect was to achieve ferroelectricity^24^. On the experimental front, however, the finite conductivity of manganites and the previously lacking evidence for the intermediate phase remained significant hurdles^23,25,36^. Our analysis provides evidence for broken inversion symmetry in the intermediate phase, which not only confirms previous theoretical proposals but also paves the way to achieving ferroelectricity if the polar state is stabilized throughout the sample. To achieve that, one approach inspired by our observations is to enhance the *Q*_*AP*_ amplitude which in turn would favor the polar intermediate phase over the centrosymmetric site-centered phase. Such manipulation could be achieved via elastic strain and rare-earth substitution which were found to modulate the amplitude of ${{{\Gamma }}}_{2}^{-}$ distortions in related theoretical calculations^41^.

Our study shows that cryo-STEM is an emerging methodology for probing complex electronic ordering phenomena through the important but often neglected lens of the lattice degrees of freedom. Using this approach, we discovered the coexistence of distinct charge-order configurations including a pure site-centered phase and a more exotic intermediate phase that breaks inversion symmetry. We also revealed that the stability of these different configurations depends on unique lattice couplings. Such a rich electronic and structural landscape should be relevant to other charge-ordered systems, including cuprates and nickelates where the exact microscopic arrangement associated with various electronic instabilities remains under intense scrutiny. Similar to the current study, microscopic insights may be achieved through direct, real space visualizations that can spatially disentangle different ordering models and characterize the intra-unit-cell structure and symmetry in detail.

## Supplementary information

Supplementray Information

## Data Availability

The data that support the findings of this work are available from PARADIM, a National Science Foundation Materials Innovation Platform (10.34863/bg5n-4s68).

## References

[1] Tranquada J, Sternlieb B, Axe J, Nakamura Y, Uchida S (1995). Evidence for stripe correlations of spins and holes in copper oxide superconductors. Nature.

[2] Mori S, Chen C, Cheong S-W (1998). Pairing of charge-ordered stripes in (La,Ca)MnO_3_. Nature.

[3] Abbamonte P (2004). Crystallization of charge holes in the spin ladder of Sr_14_Cu_24_O_41_. Nature.

[4] Sasaki S (2017). Crystallization and vitrification of electrons in a glass-forming charge liquid. Science.

[5] Chang J (2012). Direct observation of competition between superconductivity and charge density wave order in YBa_2_Cu_3_O_6.67_. Nature Physics.

[6] Li L (2016). Controlling many-body states by the electric-field effect in a two-dimensional material. Nature.

[7] Gao M (2013). Mapping molecular motions leading to charge delocalization with ultrabright electrons. Nature.

[8] Milward G, Calderon M, Littlewood P (2005). Electronically soft phases in manganites. Nature.

[9] Comin R (2015). Broken translational and rotational symmetry via charge stripe order in underdoped YBa_2_Cu_3_O_6+*y*_. Science.

[10] Comin R (2015). Symmetry of charge order in cuprates. Nat. Mater..

[11] Achkar A (2016). Orbital symmetry of charge-density-wave order in La_1.875_Ba_0.125_CuO_4_ and YBa_2_Cu_3_O_6.67_. Nat. Mater..

[12] Zhao H (2019). Charge-stripe crystal phase in an insulating cuprate. Nat. Mater..

[13] Tranquada, J., Buttrey, D., Sachan, V. & Lorenzo, J. Simultaneous ordering of holes and spins in La_2_NiO_4.125_. *Phys. Rev. Lett.* **73**, 1003 (1994). 10.1103/PhysRevLett.73.1003 10057595

[14] Uehara M, Mori S, Chen CH, Cheong SW (1999). Percolative phase separation underlies colossal magnetoresistance in mixed-valent manganites. Nature.

[15] Khomskii, D. *Transition Metal Compounds* (Cambridge University Press, 2014).

[16] McMahon, C. et al. Orbital symmetries of charge density wave order in YBa_2_Cu_3_O_6+x_. *Sci. Adv.* **6**, 45 (2020). 10.1126/sciadv.aay0345 PMC7673704 33158874

[17] Tokura Y, Nagaosa N (2000). Orbital physics in transition-metal oxides. Science.

[18] Goodenough JB (1955). Theory of the role of covalence in the perovskite-type manganites [La,M(II)]MnO_3_. Phys. Rev..

[19] Radaelli PG, Marezio M, Cheong S-W (1997). Charge, orbital, and magnetic ordering in La_0.5_Ca_0.5_MnO_3_. Phys. Rev. B.

[20] Goff R, Attfield J (2004). Charge ordering in half-doped manganites. Phys. Rev. B.

[21] Daoud-Aladine A, Rodriguez-Carvajal J, Pinsard-Gaudart L, Fernandez-Diaz M, Revcolevschi A (2002). Zener polaron ordering in half-doped manganites. Phys. Rev. Lett..

[22] Rodriguez EE, Proffen T, Llobet A, Rhyne JJ, Mitchell JF (2005). Neutron diffraction study of average and local structure in La_0.5_Ca_0.5_MnO_3_. Phy. Rev. B.

[23] Jooss C (2007). Polaron melting and ordering as key mechanisms for colossal resistance effects in manganites. Proc. Natl Acad. Sci. USA.

[24] Efremov, D. V., Van Den Brink, J. & Khomskii, D. I. Bond- versus site-centred ordering and possible ferroelectricity in manganites. *Nat. Mater.* **3**, 853–856 (2004). 10.1038/nmat1236 15558036

[25] Van Den Brink J, Khomskii DI (2008). Multiferroicity due to charge ordering. J. Phys.: Condensed Matter.

[26] El Baggari I (2018). Nature and evolution of incommensurate charge order in manganites visualized with cryogenic scanning transmission electron microscopy. Proc. Natl Acad. Sci. USA.

[27] Ogimoto Y (2005). Strain-induced crossover of the metal-insulator transition in perovskite manganites. Phys. Rev. B.

[28] Lai K (2010). Mesoscopic percolating resistance network in a strained manganite thin film. Science.

[29] Kuwahara H, Tomioka Y, Asamitsu A, Moritomo Y, Tokura Y (1995). A first-order phase transition induced by a magnetic field. Science.

[30] Grenier, S. et al. Resonant X-ray diffraction of the magnetoresistant perovskite Pr_0.6_Ca_0.4_MnO_3_. *Phys. Rev. B* **69**, 134419 (2004).

[31] Loudon J (2007). Valence changes and structural distortions in “charge ordered” manganites quantified by atomic-scale scanning transmission electron microscopy. Phys. Rev. Lett..

[32] Savitzky BH (2017). Bending and breaking of stripes in a charge ordered manganite. Nat. Commun..

[33] Egami T, Ishihara S, Tachiki M (1993). Lattice effect of strong electron correlation: Implication for ferroelectricity and superconductivity. Science.

[34] Khalyavin DD (2020). Emergent helical texture of electric dipoles. Science.

[35] Cheong, S.-W. & Mostovoy, M. Multiferroics: a magnetic twist for ferroelectricity. *Nat. Mater.* **6**, 13–20 (2007). 10.1038/nmat1804 17199121

[36] Lopes A (2008). New phase transition in the Pr_1−*x*_Ca_*x*_MnO_3_ system: evidence for electrical polarization in charge ordered manganites. Phys. Rev. Lett..

[37] Lunkenheimer P (2012). Multiferroicity in an organic charge-transfer salt that is suggestive of electric-dipole-driven magnetism. Nat. Mater..

[38] Senn MS, Wright JP, Attfield JP (2012). Charge order and three-site distortions in the verwey structure of magnetite. Nature.

[39] Hamidian MH (2012). Picometer registration of zinc impurity states in Bi_2_Sr_2_CaCu_2_O_8+*δ*_ for phase determination in intra-unit-cell Fourier transform STM. New J. Phys..

[40] van denBrink J, Khaliullin G, Khomskii D (1999). Charge and orbital order in half-doped manganites. Phys. Rev. Lett..

[41] Nowadnick EA, He J, Fennie CJ (2019). Coupled structural distortions, domains, and control of phase competition in polar SmBaMn_2_O_6_. Phys. Rev. B.

