# Supplementary material for: Charge order textures induced by non-linear couplings in a half-doped manganite
Source: Nat Commun. 2021 Jun 18;12:3747. doi: 10.1038/s41467-021-24026-7 (PMC8213702; doi:10.1038/s41467-021-24026-7)
Supplement: Supplementary file 1 — Supplementray Information [file 41467_2021_24026_MOESM1_ESM.pdf]

## METHODS AND SUPPLEMENTARY MATERIALS

**1. Pulsed Laser Deposition Growth** To grow $Nd_{1/2}Sr_{1/2}MnO_3$ thin films, $TiO_2$-terminated $SrTiO_3$ (110) substrates were pre-annealed at 950°C under oxygen partial pressure, p($O_2$), of $5.0 \times 10^{-6}$ Torr for 30 min. The particular substrate orientation of $SrTiO_3$, namely the (110) orientation, is necessary for the charge order phase to emerge, since rotational anisotropy is required [1–3]. Subsequently, 80-nm thick $Nd_{1/2}Sr_{1/2}MnO_3$ films were grown on top using a 248-nm KrF excimer laser. During the thin film deposition, the growth temperature was maintained at 825°C under p($O_2$) of $2.0 \times 10^{-3}$ Torr, using 0.40 J cm$^{-2}$ laser fluence and 11 mm$^2$ spot size.

**2. Magnetization** The magnetization data were measured using a superconducting quantum interference device (SQUID) magnetometer with an in-plane magnetic field. The sample was cooled down from room temperature to 10 K in zero external field, and its magnetization was measured in a 1000 Oe field while warming.

**3. Scanning transmission electron microscopy & data analysis**

**Experimental details.** Thin electron transparent samples were fabricated on a FEI Strata 400 focused ion beam using the standard liftout method with the stage tilted by 45°. The stage tilt enabled preparation of TEM specimens in the correct crystal orientation, as the films are grown on (110)-oriented $SrTiO_3$ substrates. High-angle annular dark-field (HAADF) STEM imaging was performed in an aberration-corrected FEI Titan Themis microscope operating at 300 kV and 50 pA probe current. The convergence semi-angle was 21.4 mrad and the inner collection angle was 68 mrad. Cryogenic STEM measurements were performed using a Gatan double-tilt side-entry liquid nitrogen TEM holder (Model 636) with a base temperature of 93 K. We acquired multiple fast-acquisition images (0.5 or 1 $\mu s$ per pixel and 1024×1024 pixels per image) to overcome sample drift which occurs at low temperature, and aligned the image series using a rigid registration method optimized for noisy images [4].

**Characterization of epitaxial film quality and fluctuations.** *Room temperature data.* The 80 nm epitaxial $Nd_{1/2}Sr_{1/2}MnO_3/SrTiO_3$ thin film is characterized using HAADF-STEM. Supplementary Fig. 1a shows a low magnification image that highlights the epitaxial

growth of the film, with the substrate appearing dimmer than the film due to the atomic number contrast in HAADF-STEM. At room temperature, no superlattice peaks associated with charge ordering are observed in the Fourier transform of STEM data (Supplementary Fig. 2), in agreement with the magnetization data which shows that charge order occurs below 150 K. We note the presence of broad superlattice peaks (Supplementary Fig.2b, green circles) which occur along the pseudocubic direction at $\mathbf{Q}^{\mathrm{cat}} = (\frac{1}{2}, 0, 0)_{\mathrm{pc}}$. These peaks exist above and below the charge ordering transition temperature and are associated with local short-range A-site (Nd/Sr) cation ordering.

*Crystal twins.* Structural inhomogeneities in bulk crystals, such as crystal twins and chemical fluctuations, often impede, or at the very least complicate, diffraction experiments since they require complex models or assumptions for structural refinements [5]. Real space imaging, on the other hand, allows us to isolate and characterize local structural or chemical fluctuations in the manganite, from the occurrence of crystal and charge order domains to short-range cation ordering. Supplementary Figure 3a shows a 40 nm region of the film in which we observe superlattice peaks along two orthogonal directions, $\mathbf{Q}_1^{CO}$ and $\mathbf{Q}_2^{CO}$, in the Fourier transform amplitude (Supplementary Fig. 3e). In the case of bi-directional order, it is important to determine whether the modulations are overlapping or spatially separated [6, 7]. By Fourier filtering charge order superlattice peaks near the (200) and (002) Bragg reflections, we obtain the local amplitude of the modulation along each direction, $A_\alpha(\mathbf{r})$ with $\alpha = 1, 2$. We define a local charge-order unidirectionality parameter

$$\Sigma^{\mathrm{CO}}(\mathbf{r}) = [A_1(\mathbf{r}) - A_2(\mathbf{r})]/[A_1(\mathbf{r}) + A_2(\mathbf{r})]$$

which determines whether the ordering pattern in locally unidirectional (positive for the $\mathbf{Q}_2^{CO}$ direction, negative for the $\mathbf{Q}_1^{CO}$ direction and close to zero for overlapped). The two charge order domains are separated by a well-defined boundary (Supplementary Fig. 3d), suggestive of a crystal twin effect rather than anomalous competition between unidirectional order parameters as seen in a previous study on a related system [7]. Indeed, we observe a splitting of crystalline Bragg peaks which indicates the presence of orthorhombic twins (Supplementary Fig. 3c). A map of the local crystal unidirectionality parameter, $\Sigma^{\mathrm{Crystal}}(\mathbf{r})$, determined using the split $(\bar{4}0\bar{2})$ Bragg peaks shows that it correlates with the charge order domains (Supplementary Fig. 3e). The crystal twinning, and hence the charge order twinning, is likely due to the relaxation of the epitaxial film since it occurs along the growth

direction.

**Mapping primary displacements.** The method for extracting periodic lattice displacements and rigorous numerical tests are described in great detail in previous work [7, 8]. To briefly summarize the method, we canvas the low temperature Fourier transform amplitude for all $\mathbf{Q}^{CO} = (1/2, 0, 0)$ superlattice peaks associated with charge ordering and damp their amplitude to the background level. We then apply an inverse Fourier transform, generating a reference image in which the modulation is removed. The positions of the atomic columns are determined by fitting 2D Gaussian functions to STEM images [7–9]. Fitting and subtracting the lattice positions in the image pair, namely the original image and the reference image, resolves the structural modification which generates the superlattice peaks at low temperature. The structural response corresponds to X1 displacements (see Methods section 4).

**Ruling out stacking of site-centered domains.** STEM imaging is a projection technique which averages out structural information along the beam direction. We must therefore address the question of whether intermediate displacements are coherent throughout the volume or simply emerge from stacked, shifted domains of site-centered order. In the latter case, Mn columns will consist of a combination of distorted Mn atoms and non-distorted Mn atoms. Due to channeling effects in HAADF-STEM, the incoherent stacking of Mn atoms would result in distorted atomic column shapes and reduced intensity [10–13]. Supplementary Fig. 5a shows a transition region where both site and intermediate orders appear. The small transition region ensures similar sample thickness. Gaussian fits of the Mn atomic columns directly yield the intensity and size parameters on each column; across the two charge order ground states, we observe no significant, systematic variation of either the column intensities or the column sizes (Supplementary Figs. 5b and c), indicating that in both cases Mn atoms are stacked coherently along the beam direction.

We can further confirm that the intermediate phase is a bona-fide ground state by comparing secondary structural responses within the site and intermediate regions. The displacements in the intermediate phase cannot emerge from simple stacking of those in the site-centered phase. For instance, we mapped the $\Gamma_2^-$ displacement pattern in the two regions (see Methods section 4). The site-centered phase shows suppressed and disordered $\Gamma_2^-$ displacements whereas the intermediate phase has coherent and prominent $\Gamma_2^-$ displacements (Supplementary Fig. 8). Incoherent stacking of the disordered displacements cannot a yield

well-defined, periodic displacement pattern in projection. Therefore, the X1 displacements in the intermediate phase are intrinsic and coherent along the imaging direction and do not reflect a stacking of phase-shifted domains of site-centered displacements.

**Mapping secondary displacements.** The $\Gamma_2^-$ mode is a zone-center mode of the orthorhombic *Pnma* cell and can be extracted using the same method if applied to the relevant peaks [7, 8]. To determine these peaks, we note that the $\Gamma_2^-$ modes (antipolar displacements of Mn atoms) correspond to zone-boundary modes of the cubic cell (Supplementary Fig. 7, dashed red square), therefore they have well defined and finite $\mathbf{q}$ peaks in the Fourier transform amplitude (Supplementary Fig. 7a, pink circles). Indeed, these modes' periodicity is encoded in the $\mathbf{q} = (1, 0, 0)$-type Fourier peaks (Supplementary Fig. 7, pink circles). To extract these displacements, we generate a reference image by removing the contribution of these particular peaks.

The obtained displacements are shown in Supplementary Fig. 7c (full field of view) and e (zoom-in). The projection of the displacements along the $\Gamma_2^-$ distortion mode (see Methods section 4 for details about this mode and Supplementary Fig. 7d for the displacement pattern) is shown in Supplementary Fig. 7f.

Over the full field of view, the amplitude and coherence of this mode fluctuates in lockstep with the fluctuation in X1 displacements (Supplementary Fig. 8). In the site-centered region, $\Gamma_2^-$ displacements are disordered and weak (Supplementary Fig. 8c and d). However, they are strong and coherent in the intermediate region (Supplementary Fig. 8e and f). As argued in the Landau theory (Methods section 4), the presence of the $\Gamma_2^-$ mode enhances the tendency to have X1 displacements on both Mn sublattices, stabilizing intermediate or bond-centered orders.

**Fourier transforms.** The complex-valued Fourier transforms, $\mathfrak{F}(\mathbf{q})$, in Figure 3 of the main text are taken from local regions with site order and intermediate order. These regions are shown in Supplementary Fig. 8. Each region is multiplied by a 2D Hann window in order to removes contributions of sharp edges to the Fourier transforms. We then take the imaginary part of the Fourier transform, $\mathfrak{Im}\{\mathfrak{F}(\mathbf{q})\}$, which is sensitive to the odd component of the signal (image) and hence can be used to detect the breaking of inversion symmetry. The real part, on the other hand, is sensitive to the even component of the signal and cannot determine inversion symmetry breaking.

A purely odd signal will have a purely imaginary and odd $\mathfrak{F}(\mathbf{q})$ while a purely even

signal will have a purely real and even $\mathfrak{F}(\mathbf{q})$. An experimental image of a crystal structure, however, is much more complicated and has contributions from imperfect centering of the signal and phase offsets, which means that the Fourier transforms will have both real and imaginary components. Since both charge-ordered structures occur within the same image, we can focus on the prominent differences in $\mathfrak{Im}\{\mathfrak{F}(\mathbf{q})\}$ between the two models. After taking the local Fourier transforms, we extract integrated line cuts across the Bragg peaks (pseudo-cubic directions). The width of the integration window corresponds to the width of the Bragg peaks.

### 4. Landau Theory

**Subgroups.** Group theory analysis and symmetry decomposition were performed using the ISOTROPY software suite [14, 15]. First, we enumerate the possible distorted structures, or subgroups, that emerge from the high-symmetry structure in the *Pnma* phase. To double the unit cell along the [100] direction, we require a zone-boundary displacement mode with $\mathbf{q} = (\frac{1}{2}, 0, 0)$. There are only two such order parameters, with one transforming like the irreducible representation (irrep) $X1$ and the other like $X2$ (notation of Miller and Love [14, 16]). The subgroups that are generated by distinct directions of the X1 irrep are $P2_1/m$, $Pnm2_1$ and $Pm$ while those that can be generated by $X2$ are $P2_1/c$, $Pna2_1$ and $Pc$.

**Displacement patterns.** We do not observe any $X2$-type displacements in the STEM data and no previous structural refinements have adequately described the charge-ordered structure in terms of the $X2$ displacement modes or their associated subgroups [17, 18]. Thus, the $X1$ mode is the relevant order parameter. While this mode involves displacements of both cations and oxygen atoms, we will focus on the Mn atoms (sites). For the Mn atoms, the $X1$ mode appears as periodic lattice displacements which can either be longitudinal, transverse and in-plane, or transverse and out-of-the plane. Our data, as well as previous refinements, show that the transverse in-plane component dominates; therefore we represent the X1 mode in terms of the transverse in-plane component, as done in Figs. 1 and 2 in the main text.

As discussed in the main text, the $X1$ order parameter is two-dimensional with each dimension corresponding to a Mn sublattice in the *Pnma* cell. The $P2_1/m$ subgroup is generated by an X1 distortion along a single dimension ($s_1$,$s_2$=0); $Pnm2_1$ by X1 distortions along both dimensions and with equal magnitude ($s_1$,$s_2$=$s_1$); and $Pm$ by X1 distortions

along both dimensions but with different magnitude ($s_1$,$s_2 \neq s_1$).

**Energy expansion and secondary modes.** We performed the free energy expansion about the high-symmetry phase (space group *Pnma*) in terms of polynomials of the X1 order parameter along the general direction, ($s_1$,$s_2$), up to fourth order [19]. Importantly, we also include the secondary modes that are allowed by symmetry [20, 21]. The possible secondary order parameters are limited to $\Gamma$ modes with $\mathbf{q} = (0,0,0)$ with respect to the *Pnma* cell, and include $\Gamma_1^+$, $\Gamma_4^+$, $\Gamma_2^-$, and $\Gamma_3^-$. The $\Gamma_1^+$ mode is a strain or volume change which appears, by symmetry, in any phase transition. The $\Gamma_4^+$ mode is a displacement mode appearing as breathing distortion of the oxygen octahedra, which, from a chemical bond perspective, corresponds to charge localization [22, 23]. Note that this "charge localization" mode alone does not double the unit cell so it must require coupling to the X1 mode. Further, according to DFT calculations, this mode by itself raises the energy of the system and only acquires a finite but small amplitude by coupling to other modes (see Methods section 5 and ref. [23]). The $\Gamma_2^-$ mode involves predominantly oxygen displacements as well as antipolar displacements of the metal cations. Since only the cations are visible in HAADF-STEM we will focus on the Mn displacement pattern associated with the $\Gamma_2^-$ irrep. Finally, $\Gamma_3^-$ corresponds to a polar displacement mode.

The free energy will contain the usual quadratic and quartic terms for each of these listed order parameters (X1 and all the listed $\Gamma$ modes) and, more importantly, coupling terms whose form depends on the symmetry of the order parameters. The full coupling energy is given by

$$F_3 = g_{vs} Q_V (s_1^2 + s_2^2) + g_{bs} Q_B (s_1^2 - s_2^2) + g_{as} Q_{AP} s_1 s_2 + g_{bap} Q_B Q_{AP} Q_P \tag{2}$$

where $Q_V$, $Q_B$, $Q_{AP}$, $Q_P$, and ($s_1$, $s_2$) are the amplitude of the $\Gamma_1+$, $\Gamma_4^+$, $\Gamma_2^-$, $\Gamma_3^-$, and X1 distortions, respectively; and $g_{vs}$, $g_{bs}$, $g_{as}$ $g_{bap}$ are the coupling coefficients. The coefficients can be determined using DFT calculations (see Methods section 5 and Supplementary Table III). We find that $g_{bs}$, $g_{as}$ and $g_{bap}$ are negative, which means that the corresponding coupling terms lower the energy.

**Interpretation of the coupling terms.** The first coupling term, $Q_V(s_1^2 + s_2^2)$, reflects the coupling of strain and volume change to the phase transition. Moreover, the $\Gamma_1^+$ mode maintains the symmetry of the parent *Pnma* structure and does not favor a particular direction in the X1 order parameter. Its main effect is quantitative, in that it slightly alters

the coefficients of the usual $s_1/s_2$ quadratic terms in the Landau energy expansion.

The interplay between the second and third coupling terms, on the other hand, is important for determining the nature of the ground state, as discussed in the main text. The second term induces the $\Gamma_4^+$ ($Q_B \neq 0$) distortion and a ($s_1$, 0) amplitude in the X1 mode, which yields site-centered order with $P2_1/m$ symmetry. The third term induces the $\Gamma_2^-$ ($Q_{AP} \neq 0$) distortion and a ($s_1$, $s_1$) X1 amplitude, which yields bond-centered order with $Pnm2_1$ symmetry. Only when both terms occur do we obtain the intermediate phase with ($s_1$, $s_2 \neq s_1$) and $Pm$ symmetry. In this case, both $\Gamma_4^+$ and $\Gamma_2^-$ are present ($Q_B \neq 0$ and $Q_{AP} \neq 0$). Plots of the energy surfaces in the cases where (a) no coupling terms are included, (b) only the second term is non-zero, (c) only the third term is non-zero and (d) both terms are non-zero are shown in Supplementary Fig. 11.

A consequence of the intermediate phase, where $Q_B$, $Q_{AP} \neq 0$, is that the fourth term, $Q_B Q_{AP} Q_P$, can become finite if a finite polarization ($Q_P \neq 0$) appears, breaking inversion symmetry and lowering the energy. That the polar state appears due to the trilinear $Q_B Q_{AP} Q_P$ coupling term suggests that ferroelectricity, if switchable, should be categorized as hybrid improper ferroelectricity [24, 25]. This analysis thus provides an explicit mechanism for the emergence of the polar state in the intermediate phase and enhances the proposal by Efremov *et al.* [26].

Finally, we note that in the main text we mainly focused on the second and third terms in $F_3$ because they are directly related to determining the stability of site and intermediate orders. That said, the other coupling terms should be considered if a more quantitative analysis is required, especially in future studies of electric field or strain effects on charge order.

## 5. Density Functional Theory

**Computational details.** We perform density functional theory (DFT) + $U$ calculations as implemented in the Vienna *ab initio* simulation package (VASP) [27, 28] with the PBEsol exchange-correlation functional [29] and a 500 eV energy cutoff for the plane-wave basis. We perform our calculations in an orthorhombic 40 atom unit cell with dimensions $2\sqrt{2} \times 2 \times \sqrt{2}$ and choose a $4 \times 6 \times 8$ $\Gamma$-centered $k$-point grid. We use the Liechtenstein formulation of DFT + $U$ [30], where we set $U = 5$ eV and $J = 1.2$ eV. For structural relaxations, we use a 2.5 meV/Å force convergence tolerance.

The starting point of the calculation is the high-symmetry structure $P4/mmm$ (which would correspond to the cubic cell without cation order) with Nd/Sr cations ordered along the unique **b**-axis. We perform calculations with ferromagnetic (FM), A-type antiferromagnetic (A-AFM), and CE-type antiferromagnetic (CE-AFM) orders imposed. Note that for the CE-AFM case, we use an 80 atom cell with a $4 \times 6 \times 4$ $k$-point grid. The AFM-coupling in the A- and CE-AFM states is imposed along the **b**-axis. We find that the polar $P2_1ma$ structure (intermediate phase) has the lowest energy (Supplementary Table I). Note that in the DFT-calculated structures, the computational requirement for A-site (Nd/Sr) ordering changes the symmetry.

**Displacement modes.** To assess the symmetries and amplitudes of the structural distortions that contribute to the $P2_1ma$ structure, we report in Supplementary Table II a structural decomposition of the DFT-relaxed $P2_1ma$ structure into symmetry adapted modes of the $P4/mmm$ reference structure [14, 15]. Note that since we are using a $P4/mmm$ (rather than $Pnma$) reference structure here, the labelling of the irreps is different, although they represent the same physical atomic displacements. The $M_5^-$ mode, which is an octahedral tilting mode (contained in $Pnma$) together with antipolar Mn displacements, and the $\Sigma_2$ mode, which corresponds to the X1-type periodic lattice displacements, have the largest amplitudes, followed by the $M_4^+$ oxygen breathing distortion. The polar ($\Gamma_5^-$) distortion is also present. For each distortion, we use the displacement of each distinct atomic type to construct the relevant symmetry-adapted mode vector, which we use for our energy surface calculations below [23].

**Landau expansion about the $P4/mmm$ reference structure.** With the $P4/mmm$ irreps defined above, we perform the energy expansion of the free energy, $F_1$, about the high-symmetry $P4/mmm$ reference structure [21]. To fourth order, the energy is

$$\begin{aligned} F_1 &= \tfrac{1}{2}\alpha_t Q_{AP}^2 + \tfrac{1}{4}\beta_t Q_{AP}^4 \\ &+ \tfrac{1}{2}\alpha_s(s_1^2 + s_2^2) + \tfrac{1}{4}\beta_s(s_1^2 + s_2^2)^2 + \tfrac{1}{2}\gamma_s s_1^2 s_2^2 \\ &+ \tfrac{1}{2}\alpha_b Q_B^2 + \tfrac{1}{2}\alpha_p Q_P^2 \\ &+ F_3 \end{aligned} \tag{3}$$

where $F_3$ is the third-order coupling energy

$$F_3 = g_{as} Q_{AP} s_1 s_2 + g_{bs} Q_B (s_1^2 - s_2^2) + g_{bap} Q_{AP} Q_B Q_P. \tag{4}$$

Here, $Q_{AP}$, $Q_B$, $Q_P$, and ($s_1$,$s_2$) are the amplitudes of the $M_5^-$, $M_4^+$, $\Gamma_5^-$ and $\Sigma_2$ displacement modes, respectively (see Table II in Methods section for the amplitudes). Note that the coupling terms in $F_3$ have the same functional form as in equation (2) in Methods section 4.

**Determining the coupling coefficients.** We calculate energy surfaces by "freezing in" increasing amplitudes of each mode and calculating the total energy with DFT, as shown in Supplementary Fig. 9. By fitting these energy surfaces we extract the Landau expansion coefficients $\alpha_i$ and $\beta_i$ in equation (3). Note that we freeze in the two-dimensional $\Sigma_2$ order parameter along the direction (0.907, 0.421), which is the direction this order parameter takes in the DFT-relaxed $P2_1ma$ structure with A-AFM order. That is, we freeze in amplitude $Q_s = \sqrt{s_1^2 + s_2^2}$ where $s_1 = 0.907Q_s$ and $s_2 = 0.421Q_s$. For this reason, we omit the $\gamma_s$ coefficient from equation (3).

To calculate the coupling coefficients $g_{as}$ and $g_{bs}$ in equation (4), we fix the $\Sigma_2$ distortion amplitude to the value obtained in the DFT-relaxed structure ($Q_s$ = 0.68 Å), freeze in the $M_5^-$ distortion ($M_4^+$ breathing distortion), and then fit the resulting energy surface (Supplementary Fig. 10a,b). To calculate the trilinear $g_{tbp}$ coefficient, we fix the $M_5^-$ and $M_4^+$ amplitudes to the values in the DFT-relaxed structure and freeze in the polar mode (Supplementary Fig. 10c). The resulting coupling coefficients are reported in Supplementary Table III.

The $\alpha_s$, $\alpha_b$, $\alpha_p$ coefficients are all positive which means that the X1-like displacements, the breathing distortion, and the polar distortion by themselves do not lower the energy. The breathing distortion and the polar distortion significantly raise the energy so we limited the energy expansion to the second powers of $Q_B$ and $Q_P$. Meanwhile, the coupling coefficients $g_{as}$, $g_{bs}$ and $g_{bap}$ are negative, which means that the coupling terms lower the energy of the system by non-linearly inducing distortions that by themselves are energetically unfavorable. The energy surfaces and line plots in Supplementary Fig. 11 and Fig. 4 of the main text are generated from supplementary equations (3)-(4) and Supplementary Table III. These results show that non-linear lattice coupling are essential for understanding the formation of the charge-ordered states.

---

[1] Nakamura, M., Ogimoto, Y., Tamaru, H., Izumi, M., Miyano, K. Phase control through anisotropic strain in $Nd_{0.5}Sr_{0.5}MnO_3$ thin films. *Applied Physics Letters* **86**, 1–3 (2005).

[2] Ogimoto, Y., Nakamura, M., Takubo, N., Tamaru, H., Izumi, M., Miyano, K. Strain-induced crossover of the metal-insulator transition in perovskite manganites. *Physical Review B* **71**, 060403 (2005).

[3] Lai, K., Nakamura, M., Kundhikanjana, W., Kawasaki, M., Tokura, Y., Kelly, M. A., Shen, Z.-X. Mesoscopic percolating resistance network in a strained manganite thin film. *Science* **329**, 190–193 (2010).

[4] Savitzky, B., El Baggari, I., Clement, C., Waite, E., Goodge, B., Baek, D., Sheckelton, J., Pasco, C., Nair, H., Schreiber, N., Hoffman, J., Admasu, A., Kim, J., Cheong, S.-W., Bhattacharya, A., Schlom, D., McQueen, T., Hovden, R., Kourkoutis, L. Image registration of low signal-to-noise cryo-STEM data. *Ultramicroscopy* **191**, 56–65 (2018).

[5] Woodward, P., Cox, D., Vogt, T., Rao, C., Cheetham, A. Effect of Compositional Fluctuations on the Phase Transitions in $(Nd_{1/2}Sr_{1/2})MnO_3$. *Chemistry of Materials* **11**, 3528–3538 (1999).

[6] Comin, R., Sutarto, R., daSilva Neto, E. H., Chauviere, L., Liang, R., Hardy, W. N., Bonn, D. A., He, F., Sawatzky, G. A., Damascelli, A. Broken translational and rotational symmetry via charge stripe order in underdoped $YBa_2Cu_3O_{6+y}$. *Science* **347**, 1335–1339 (2015).

[7] Savitzky, B. H., El Baggari, I., Admasu, A. S., Kim, J., Cheong, S.-W., Hovden, R., Kourkoutis, L. F. Bending and breaking of stripes in a charge ordered manganite. *Nature Communications* **8**, 1–6 (2017).

[8] El Baggari, I., Savitzky, B. H., Admasu, A. S., Kim, J., Cheong, S.-W., Hovden, R., Kourkoutis, L. F. Nature and evolution of incommensurate charge order in manganites visualized with cryogenic scanning transmission electron microscopy. *Proceedings of the National Academy of Sciences* **115**, 1445–1450 (2018).

[9] Yankovich, A. B., Berkels, B., Dahmen, W., Binev, P., Sanchez, S. I., Bradley, S. A., Li, A., Szlufarska, I., Voyles, P. M. Picometre-precision analysis of scanning transmission electron microscopy images of platinum nanocatalysts. *Nature Communications* **5**, 4155 (2014).

[10] Perovic, D., Rossouw, C., Howie, A. Imaging elastic strains in high-angle annular dark field scanning transmission electron microscopy. *Ultramicroscopy* **52**, 353–359 (1993).

[11] Hillyard, S., Silcox, J. Detector geometry, thermal diffuse scattering and strain effects in ADF STEM imaging. *Ultramicroscopy* **58**, 6–17 (1995).

[12] Fitting, L., Thiel, S., Schmehl, A., Mannhart, J., Muller, D. A. Subtleties in ADF imaging and spatially resolved EELS: A case study of low-angle twist boundaries in $SrTiO_3$. *Ultrami-*

*croscopy* **106**, 1053–1061 (2006).

[13] Haruta, M., Kurata, H., Komatsu, H., Shimakawa, Y., Isoda, S. Effects of electron channeling in HAADF-STEM intensity in $La_2CuSnO_6$. *Ultramicroscopy* **109**, 361–367 (2009).

[14] Stokes, H. T., Hatch, D. M., Campbell, B. J. ISOTROPY Software Suite (`iso.byu.edu`).

[15] Stokes, H. T., Hatch, D. M., Campbell, B. J., Tanner, D. E. ISODISPLACE: A web-based tool for exploring structural distortions. *Journal of Applied Crystallography* **39**, 607–614 (2006).

[16] Miller, S. C., Love, W. F. (1967) *Tables of irreducible representations of space groups and co-representations of magnetic space groups.* Pruett Press.

[17] Radaelli, P. G., Marezio, M., Cheong, S.-W. W. Charge, orbital, and magnetic ordering in $La_{0.5}Ca_{0.5}MnO_3$. *Physical Review B* **55**, 3015–3023 (1997).

[18] Daoud-Aladine, A., Rodriguez-Carvajal, J., Pinsard-Gaudart, L., Fernandez-Diaz, M., Revcolevschi, A. Zener polaron ordering in half-doped manganites. *Physical Review Letters* **89**, 097205 (2002).

[19] Hatch, D. M., Stokes, H. T. Invariants: program for obtaining a list of invariant polynomials of the order-parameter components associated with irreducible representations of a space group. *Journal of Applied Crystallography* **36**, 951–952 (2003).

[20] Hatch, D. M., Stokes, H. T. Complete listing of order parameters for a crystalline phase transition: A solution to the generalized inverse Landau problem. *Physical Review B* **65**, 014113 (2001).

[21] Stokes, H. T., Hatch, D. M. Copl: program for obtaining a complete list of order parameters for a group-subgroup crystalline phase transition. *Journal of Applied Crystallography* **35**, 379–379 (2002).

[22] Brown, I. Chemical and steric constraints in inorganic solids. *Acta Crystallographica Section B: Structural Science* **48**, 553–572 (1992).

[23] Nowadnick, E. A., He, J., Fennie, C. J. Coupled structural distortions, domains, and control of phase competition in polar $SmBaMn_2O_6$. *Physical Review B* **100**, 195129 (2019).

[24] Benedek, N. A., Fennie, C. J. Hybrid improper ferroelectricity: a mechanism for controllable polarization-magnetization coupling. *Physical Review Letters* **106**, 107204 (2011).

[25] Pitcher, M. J., Mandal, P., Dyer, M. S., Alaria, J., Borisov, P., Niu, H., Claridge, J. B., Rosseinsky, M. J. Tilt engineering of spontaneous polarization and magnetization above 300 K in a bulk layered perovskite. *Science* **347**, 420–424 (2015).

[26] Efremov, D. V., Van Den Brink, J., Khomskii, D. I. Bond- Versus site-centred ordering and possible ferroelectricity in manganites. *Nature Materials* **3**, 853–856 (2004).

[27] Kresse, G., Hafner, J. Ab initio molecular dynamics for liquid metals. *Physical Review B* **47**, 558 (1993).

[28] Kresse, G., Joubert, D. From ultrasoft pseudopotentials to the projector augmented-wave method. *Physical Review B* **59**, 1758–1775 (1999).

[29] Perdew, J. P., Ruzsinszky, A., Csonka, G. I., Vydrov, O. A., Scuseria, G. E., Constantin, L. A., Zhou, X., Burke, K. Restoring the density-gradient expansion for exchange in solids and surfaces. *Physical Review letters* **100**, 136406 (2008).

[30] Liechtenstein, A., Anisimov, V., Zaanen, J. Density-functional theory and strong interactions: Orbital ordering in Mott-Hubbard insulators. *Physical Review B* **52**, R5467 (1995).

Supplementary Table I. Selected structural phases of 010 cation-ordered $NdSrMn_2O_6$. For each subgroup and magnetic order, structural relaxations were performed with DFT + $U$. Energies are reported for a 40 atom cell. The first two columns indicate the direction of the $M_5^-$ and $\Sigma_2$ irreps in each subgroup.

| $M_5^-$ | $\Sigma_2$ | Space group | Magnetic state | Energy [meV/f.u.] |
|---|---|---|---|---|
| - | - | $P4/mmm$ | A-AFM | 0.0 |
| - | - | $P4/mmm$ | FM | -19.5 |
| ($a$,0) | - | $Pmam$ | A-AFM | -121.3 |
| ($a$,0) | - | $Pmam$ | FM | -126.6 |
| ($a$,$a$) | - | $Cmmm$ | A-AFM | -133.6 |
| ($a$,$a$) | - | $Cmmm$ | FM | -138.2 |
| - | ($a$,$b$,0,0) | $P2_1ma$ | CE-AFM | -161.3 |
| - | ($a$,$b$,0,0) | $P2_1ma$ | A-AFM | -154.7 |

Supplementary Table II. Decomposition of the DFT+$U$-relaxed intermediate phase ($P2_1ma$) structure into symmetry adapted modes of $P4/mmm$. Here 010 cation order and A-type AFM order is imposed in the calculations. Amplitudes are given in units of Å for the 40 atom unit cell. The relaxed lattice vectors for the $P2_1ma$ cell are $a$=11.001 Å, $b$ = 7.468 Å, and $c$ = 5.445 Å. The description shows the corresponding irrep in the case of no cation order (Methods section 4).

| Irrep | Wavevector | Amplitude (Å) | Description |
|---|---|---|---|
| $M_5^-$ | $(\frac{1}{2}, \frac{1}{2}, 0)$ | 1.23 | Antipolar Mn displacements ($\Gamma_2^-$) |
| $M_4^+$ | $(\frac{1}{2}, \frac{1}{2}, 0)$ | 0.17 | Oxygen breathing distortion ($\Gamma_4^+$) |
| $\Sigma_2$ | $(\frac{1}{4}, \frac{1}{4},0)$ | (0.907, 0.421) | X1-like periodic displacements |
| $\Gamma_5^-$ | (0,0,0) | 0.05 | Polar distortion ($\Gamma_5^-$) |

Supplementary Table III. Parameters from fitting the free-energy expansion in equations (3)-(4) to the DFT-calculated energy surfaces shown in Supplementary Fig. 9.

| | | |
|---|---|---|
| $\alpha_t = -0.31$ eV/Å$^2$ | $\beta_t = 0.28$ eV/Å$^4$ | $g_{as} = -0.31$ eV/Å$^3$ |
| $\alpha_s = 0.17$ eV/Å$^2$ | $\beta_s = 1.01$ eV/Å$^4$ | $g_{bs} = -2.14$ eV/Å$^3$ |
| $\alpha_b = 2.72$ eV/Å$^2$ | | $g_{bap} = -0.24$ eV/Å$^3$ |
| $\alpha_p = 0.88$ eV/Å$^2$ | | |

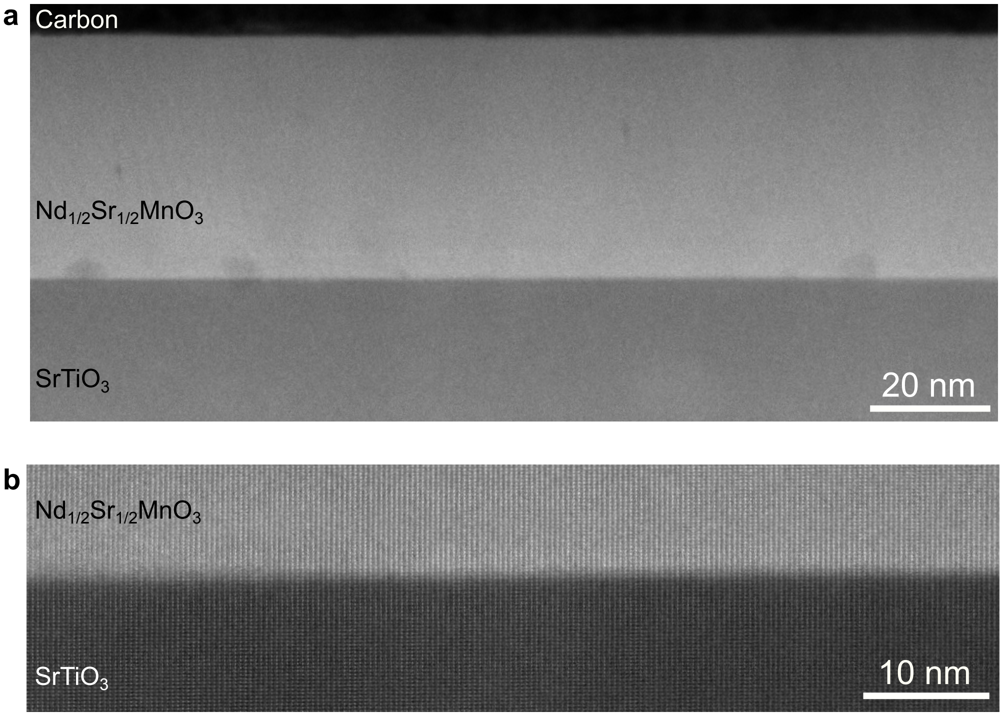


Supplementary Fig. 1. Thin film growth of $Nd_{1/2}Sr_{1/2}MnO_3$. **a**, Overview HAADF-STEM image of the sample. A 80 nm thick film of $Nd_{1/2}Sr_{1/2}MnO_3$ is grown on a $SrTiO_3$ substrate using pulsed laser deposition. **b**, Higher magnification STEM image showing the (110) orientation of the $SrTiO_3$ substrate and high-quality epitaxial growth of the $Nd_{1/2}Sr_{1/2}MnO_3$ film.

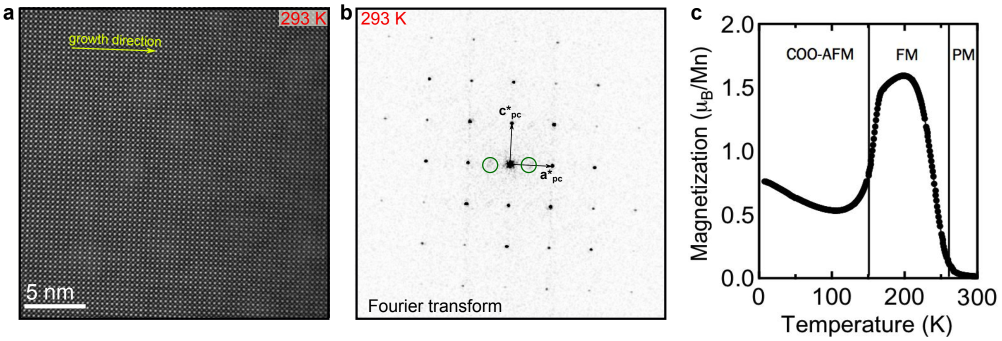


Supplementary Fig. 2. Room temperature STEM data and temperature-dependent magnetization. **a**, HAADF STEM image of the $Nd_{1/2}Sr_{1/2}MnO_3$ thin film at room temperature. **b**, At this temperature, the Fourier transform amplitude shows no superlattice peaks associated with charge ordering, in agreement with the magnetization data. Broad peaks associated with short-range cation order along the pseudocubic $\mathbf{a}^*_{pc}$-direction are present (green circles). These peaks can also be seen in the low temperature data shown in Fig. 1 of the main text. **c**, Magnetization data showing a paramagnetic to ferromagnetic (FM) transition at 260 K and a FM to charge-ordered AFM transition at 150 K. The data is consistent with previous epitaxial films [1–3].

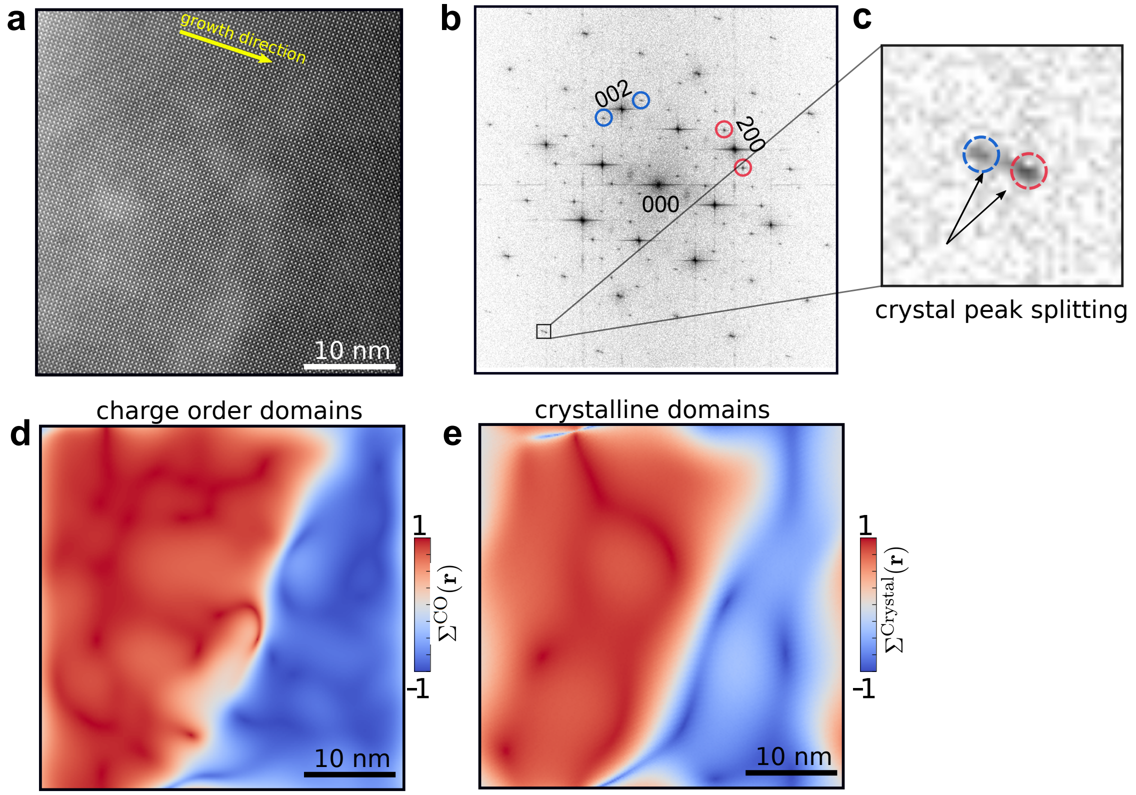


Supplementary Fig. 3. Crystal and charge-order domains are correlated. **a**, HAADF-STEM image over a large field of view (40 nm x 40 nm). **b**, Fourier transform amplitude highlighting orthogonal charge order peaks (blue/red circles). **c**, Zoom-in of the $(\bar{4}0\bar{2})$ crystal Bragg peak. A clear splitting is evident indicating the presence of crystalline orthorhombic twins. **d**, Local charge order unidirectionality parameter, $\Sigma^{\mathrm{CO}}(\mathbf{r})$. A positive/negative value indicates that the contribution of the red/blue-highlighted charge order peak in **b** dominates locally. **e**, Local crystal unidirectionality parameter, $\Sigma^{\mathrm{Crystal}}(\mathbf{r})$, mapped from the split $(\bar{4}0\bar{2})$ crystalline peaks (dashed circles). A positive/negative value indicates that the contribution of the red/blue-highlighted crystalline peak in **c** dominates locally. The map shows a well-defined crystalline domain boundary. Further, this boundary tracks perfectly the charge domain boundary, indicating that the observed charge order twinning is due to crystal twinning and not to local nanoscale charge order clusters or overlapping domains.

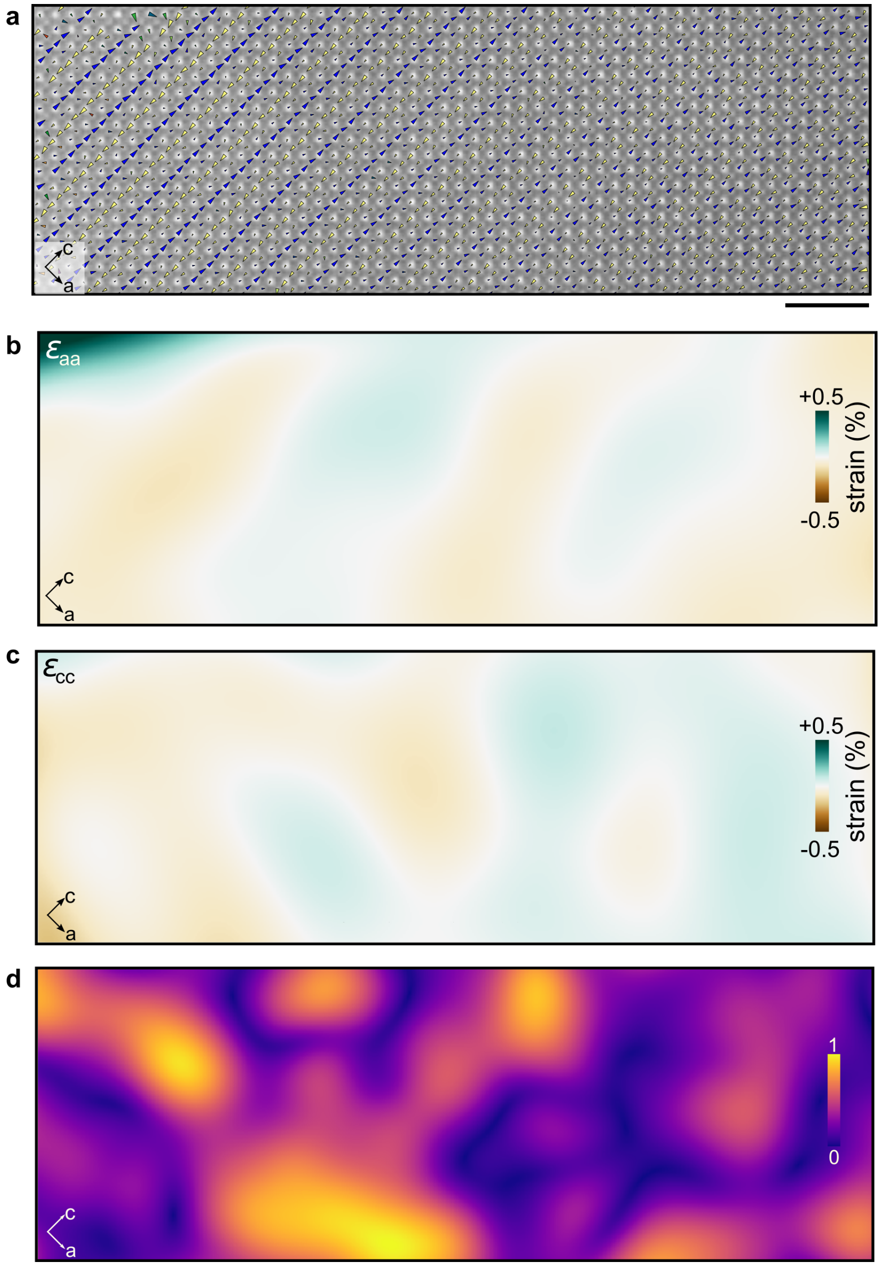


Supplementary Fig. 4. Strain and cation disorder. **a**, Map of charge order displacements showing transition from site-centered to intermediate order. **b, c**, Map of the lattice strain $\varepsilon_{aa}$ and $\varepsilon_{cc}$, respectively. The strain values are small and show little to no correlation with the nature of the charge order ground state. **d**, Real space map of the Fourier amplitude associated with short-range cation order with wavevector $\mathbf{q} = \frac{1}{2}\mathbf{a}^*_{\mathbf{pc}}$. All panels are in the same field of view and share the same scale bar. Scale bar is 2 nm.

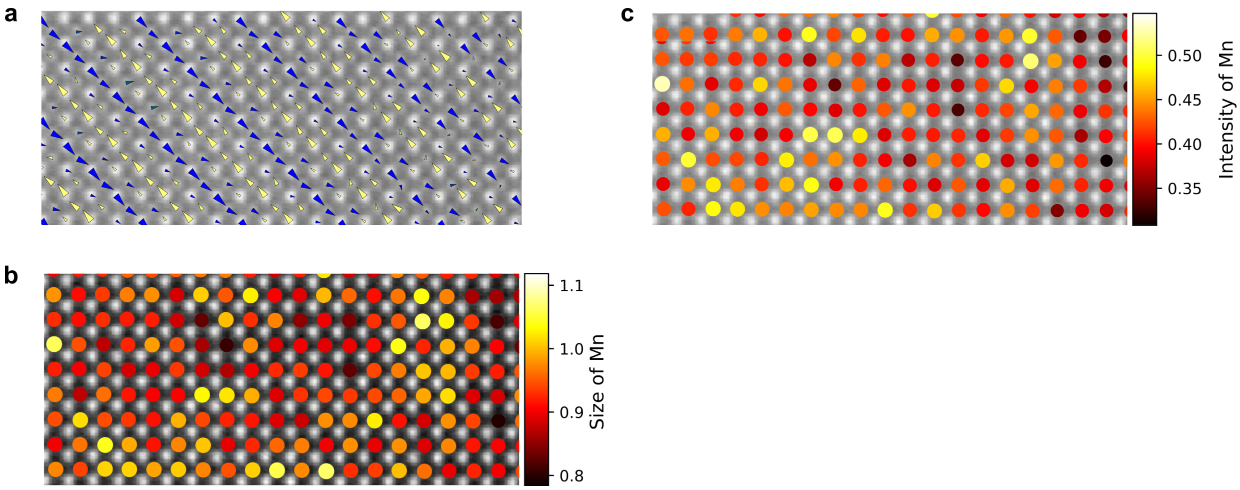


Supplementary Fig. 5. Ruling out stacking of site-centered order. **a**, Transition region containing regions with Mn displacements on one sublattice only (site-centered) and regions with Mn displacements on two sublattices. If the latter emerges from out-of-plane stacking of site-centered domains, Mn columns would contain a combination of distorted and un-distorted atoms, leading to changes in HAADF-STEM intensity due to electron beam channeling. **b,c**, Gaussian fits to Mn columns in the transition region show no systematic variations of intensity or size of Mn columns. For instance, brighter/darker columns occur in both the site-centered regions and the regions with displacements on both Mn sublattices.

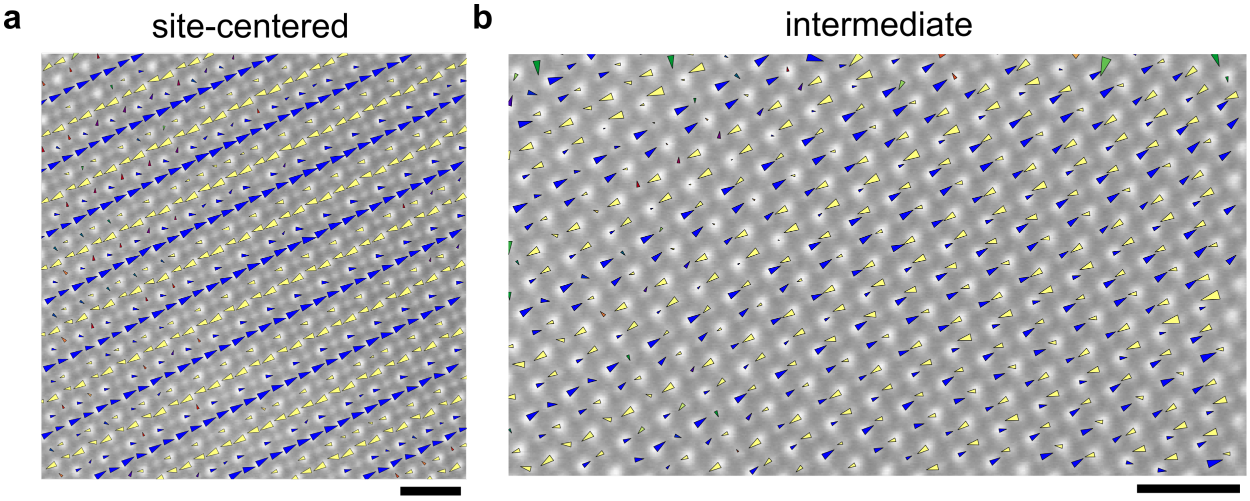


Supplementary Fig. 6. Additional data showing regions of site or intermediate order. **a,b**, Site-centered order and intermediate order in regions distinct from those in the text. Both the Mn and Nd/Sr displacements are shown here. The scale bars correspond to 1 nm.

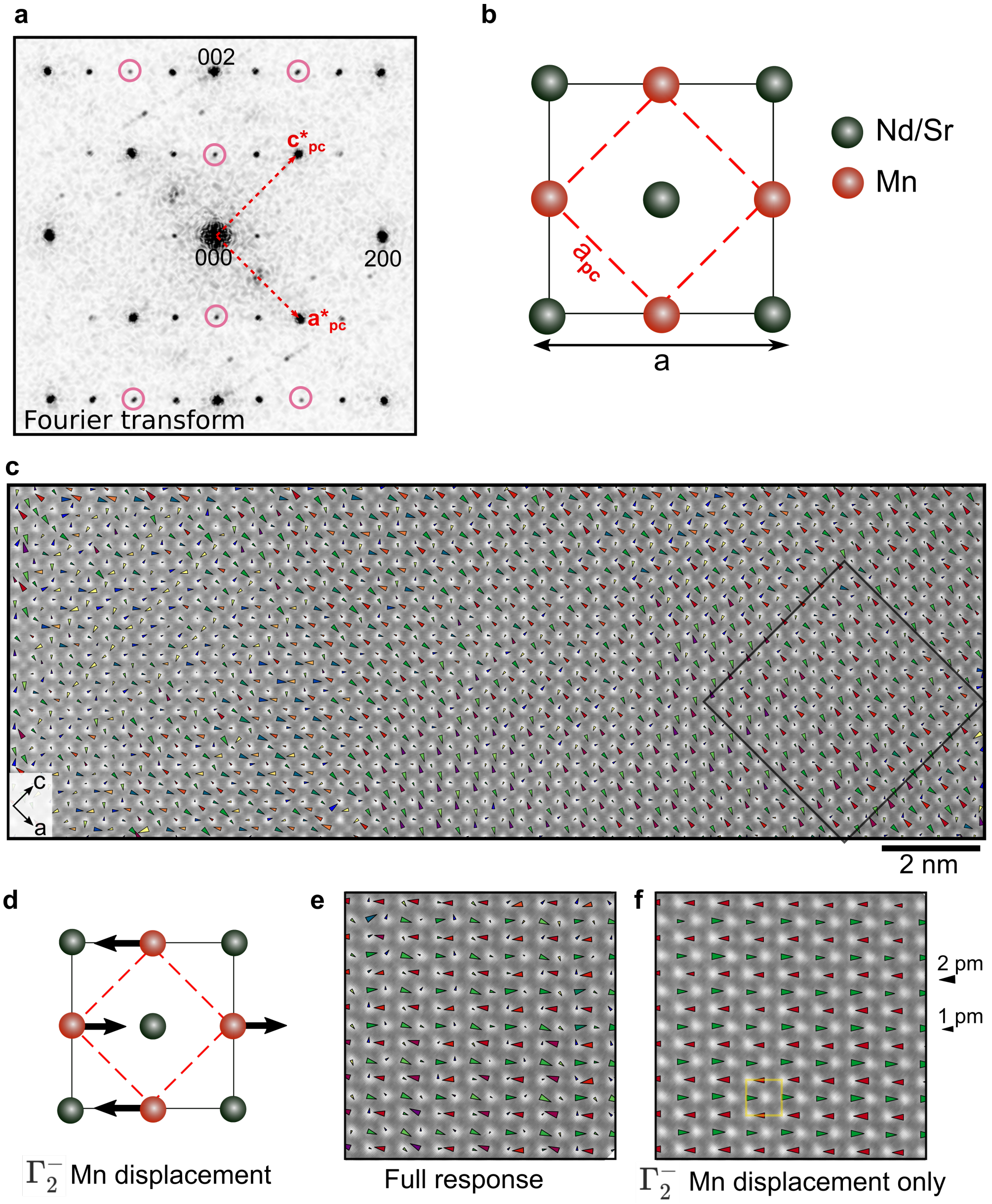


Supplementary Fig. 7. Extracting $\Gamma_2^-$ displacements. **a**, Fourier transform amplitude in the low temperature phase. Peaks associated with the $\Gamma$ modes of the orthorhombic cell are highlighted by pink circles. The indexing of the peaks assumes a *Pnma* structure. **b**, Relationship between the orthorhombic cell (black square) and the pseudocubic cell (red dashed square). **c**, Map of the lattice displacements associated with the orthorhombic peaks (pink circles in a). The Nd/Sr displacements are negligible. **d**, Displacement pattern of the $\Gamma_2^-$ mode on Mn sites. These displacements fit in the orthorhombic cell. **e**, Zoom-in of the region within the black square in **c**. **f**, Projection of the extracted Mn displacements onto the $\Gamma_2^-$ mode direction.

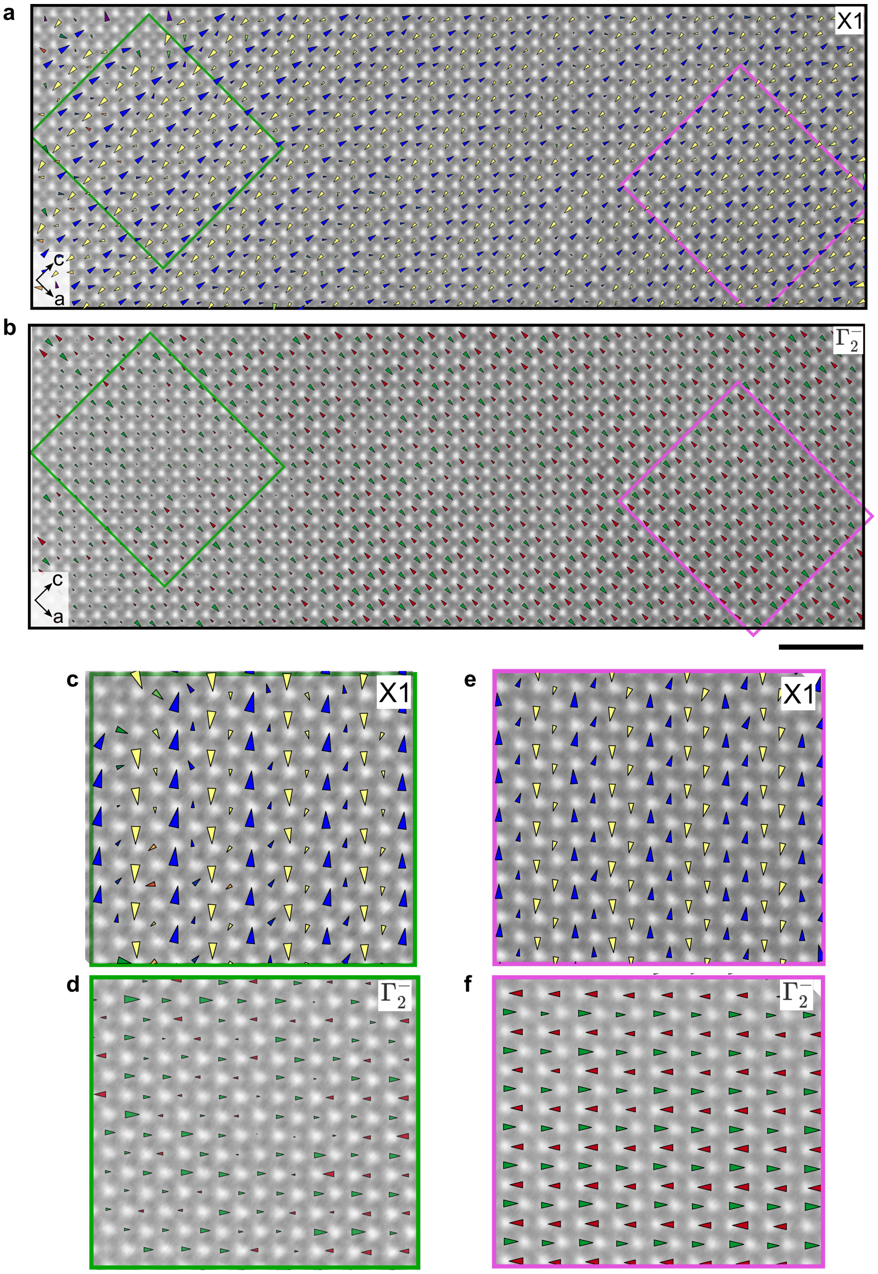


Supplementary Fig. 8. Interplay between $\Gamma_2^-$ displacements and $X1$ displacements. **a**, Large field-of-view of X1 Mn displacements that transition from a site-centered (green) state to an intermediate (purple) state. The regions marked by the rectangles span those shown in Fig. 3 in the main text. **b**, Map of $\Gamma_2^-$ Mn displacements in the same region as a. **c,d**, Map of the X1 and $\Gamma_2^-$ Mn displacements within the site-centered region. **e,f**, Map of the X1 and $\Gamma_2^-$ Mn displacements within the intermediate region. The $\Gamma_2^-$ displacements are weak and disordered within the region containing site-centered order but are strong and coherent within the region containing intermediate order, in agreement with the Landau theory prediction.

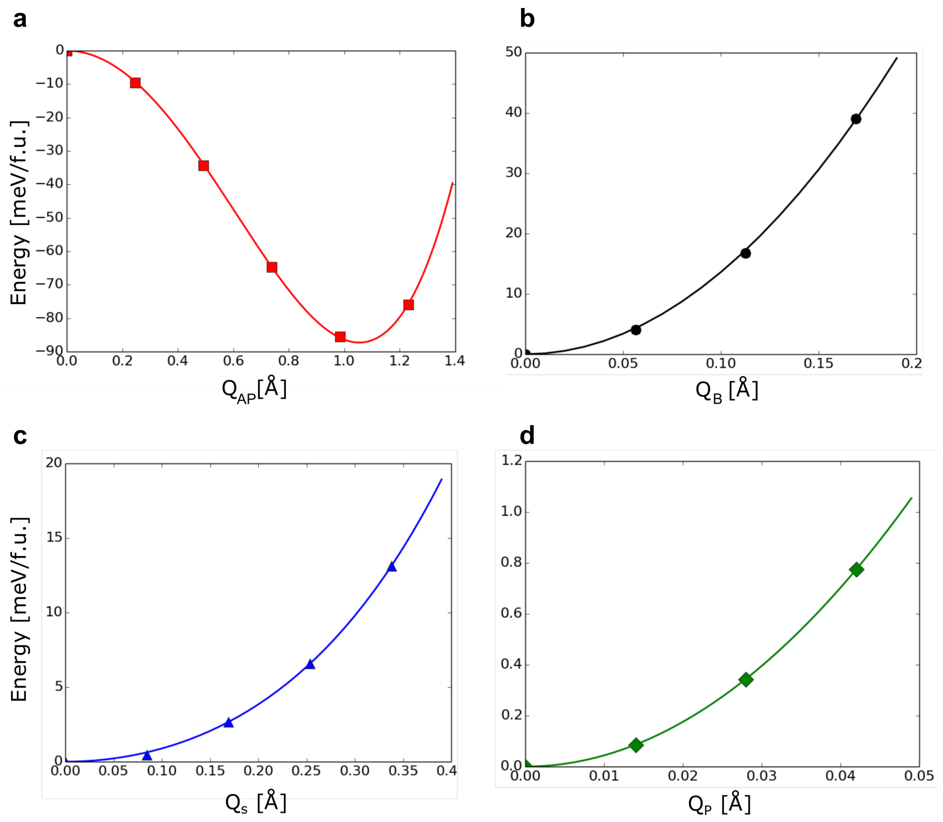


Supplementary Fig. 9. Energy surfaces calculated with DFT by freezing in **a**, the $M_5^-$ distortion ($Q_{AP}$), **b**, the $M_4^+$ breathing distortion ($Q_B$), **c**, the $\Sigma_2$ distortion ($X1$-like displacements), and **d**, the $\Gamma_5^-$ ($Q_P$) polar distortion. By fitting these energy surfaces, we obtain the coefficients in equation (3). The calculations are done in a 40 atom unit cell with A-AFM ordered along the **b**-axis.

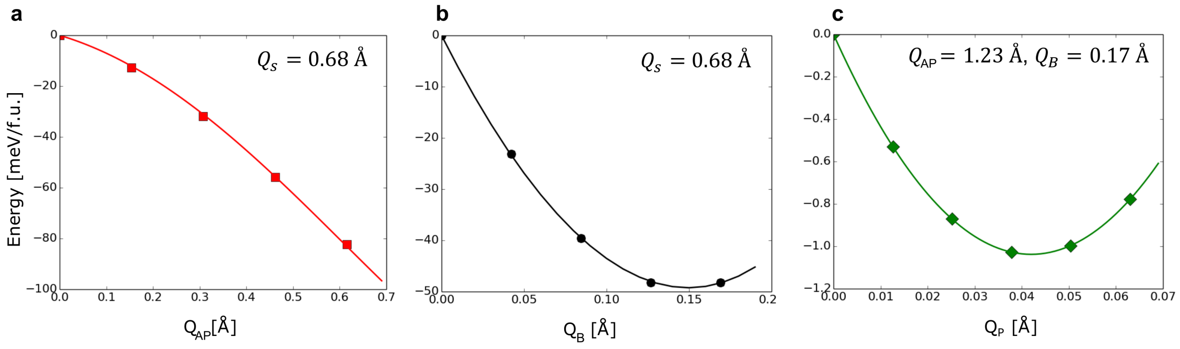


Supplementary Fig. 10. Energy surfaces used to calculate the coupling coefficients in equation (4). **a,b**, The $\Sigma_2$ (X1-like) distortion is set to a fixed amplitude and then $Q_{AP}$, $Q_B$ is frozen in. **c**, Both $Q_{AP}$ and $Q_B$ are fixed, and the polar mode $Q_P$ is frozen in.

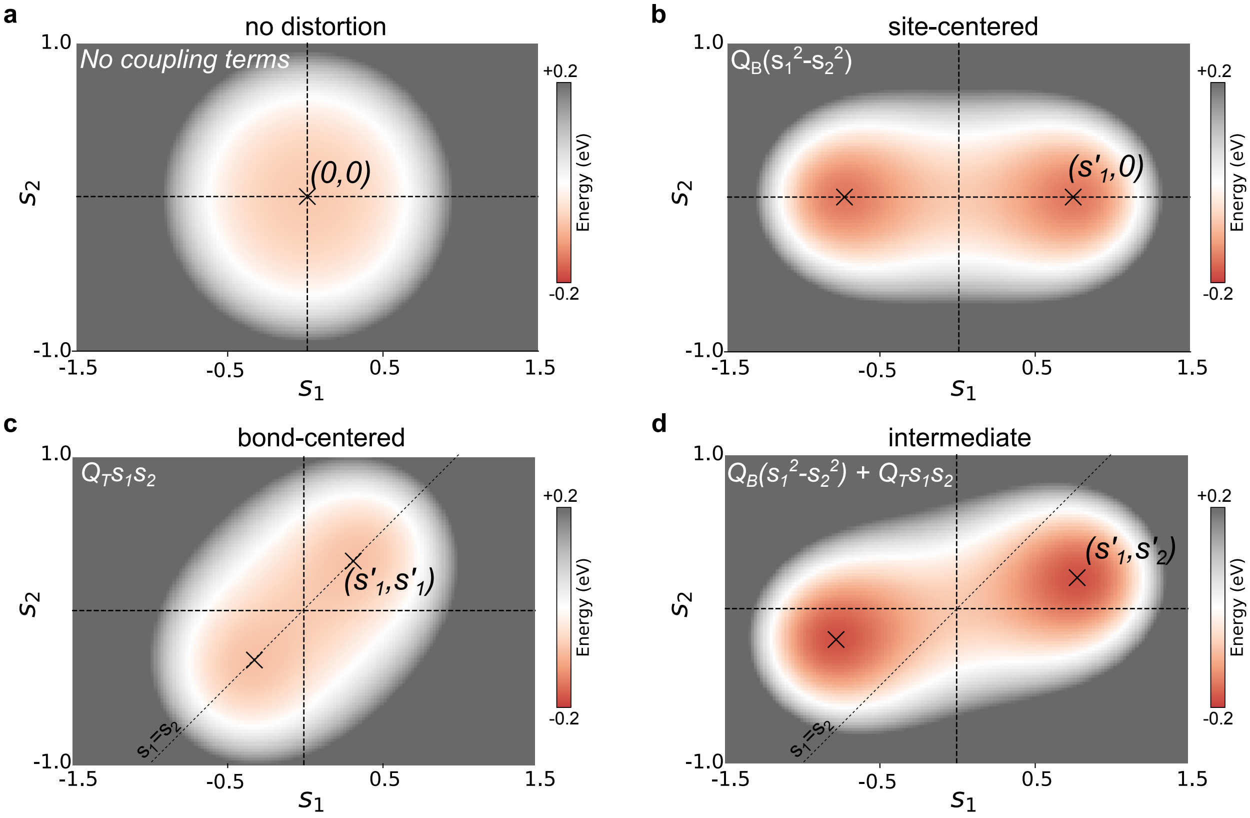


Supplementary Fig. 11. Energy surfaces based on the Landau theory. **a**, If the third-order coupling terms are removed from equation (3), the displacements associated with the charge-ordered phase do not lower the free-energy. **b**, Adding the $g_{bs}Q_B(s_1^2 - s_2^2)$ coupling term lowers the energy substantially by having a finite amplitude in the X1-like displacement order parameter. The energy is minimized (black cross) by having the X1-like amplitude equal to ($s_1'$,0) (site-centered order). **c**, By including only the $g_{as}Q_{AP}s_1s_2$ coupling term, the energy is minimized at the ($s_1'$,$s_2' = s_1'$) coordinate of the X1-like order parameter (bond-centered order). **d**, When both coupling terms are present, the energy is minimized at the ($s_1', s_2' \neq s_1'$) coordinate of the X1-like order parameter (intermediate order). We set the amplitudes of $Q_B$, $Q_{AP}$, and $Q_P$ to the values in Supplementary Table II and the coefficients to those in Supplementary Table III. The picture does not change qualitatively by varying these values as long as the sign of the coefficients remains the same. The linecuts for Fig. 4 in the main text were taken from panels b and d, along the (0,$s_2$) directions and passing though the minimum energy positions (crosses).
